# Ferroptosis Activation Scoring Model Assists in Chemotherapeutic Agents’ Selection and Mediates Cross-Talk With Immunocytes in Malignant Glioblastoma

**DOI:** 10.3389/fimmu.2021.747408

**Published:** 2022-01-19

**Authors:** Zeyu Wang, Ziyu Dai, Lifu Zheng, Binyuan Xu, Hao Zhang, Fan Fan, Xun Zhang, Xisong Liang, Zhixiong Liu, Kui Yang, Quan Cheng

**Affiliations:** ^1^ Department of Neurosurgery, Xiangya Hospital, Central South University, Changsha, China; ^2^ National Clinical Research Center for Geriatric Disorders, Xiangya Hospital, Central South University, Changsha, China; ^3^ Clinic Medicine of 5-Year Program, Xiangya School of Medicine, Central South University, Changsha, China; ^4^ Clinical Diagnosis and Therapy Center for Gliomas of Xiangya Hospital, Central South University, Changsha, China; ^5^ Department of Clinical Pharmacology, Xiangya Hospital, Central South University, Changsha, China

**Keywords:** ferroptosis, glioblastoma, mesenchymal, immunocytes, cell–cell communication

## Abstract

Gliomas are aggressive tumors in the central nervous system and glioblastoma is the most malignant type. Ferroptosis is a programmed cell death that can modulate tumor resistance to therapy and the components of tumor microenvironment. However, the relationship between ferroptosis, tumor immune landscape, and glioblastoma progression is still elusive. In this work, data from bulk RNA-seq analysis, single cell RNA-seq analysis, and our own data (the Xiangya cohort) are integrated to reveal their relationships. A scoring system is constructed according to ferroptosis related gene expression, and high scoring samples resistant to ferroptosis and show worse survival outcome than low scoring samples. Notably, most of the high scoring samples are aggressive glioblastoma subtype, mesenchymal, and classical, by calculating RNA velocity. Cross-talk between high scoring glioblastoma cells and immunocytes are explored by R package ‘celltalker’. Ligand–receptor pairs like the TRAIL or TWEAK signaling pathway are identified as novel bridges implying how ferroptosis modulate immunocytes’ function and shape tumor microenvironment. Critically, potential drugs target to high scoring samples are predicted, namely, SNX2112, AZ628, and bortezomib and five compounds from the CellMiner database. Taken together, ferroptosis associates with glioblastoma aggressiveness, cross-talk with immunocytes and offer novel chemotherapy strategy.

## Introduction

Gliomas are malignant tumors of the central nervous system (1). Histologically, gliomas can be classified into several groups, including astrocytic tumors, oligodendroglial tumors, oligoastrocytic tumors, ependymal tumors, mixed neuronal glial tumors (such as gangliogliomas), etc. WHO grade IV gliomas, also known as glioblastomas (GBMs), are the most aggressive type of gliomas with median overall survival time of GBM less than 14.6 months (2). Four subtypes of GBM (proneural, neural, classical, and mesenchymal) which were proposed by Verhhak and his team based on GBM genome characteristics have been proved that can predict GBM prognosis (3). Mesenchymal and classical GBM show more aggressive growth pattern, while proneural and neural GBM have better prognosis. Treatments like surgical removal, radiation therapy, and chemotherapy can slow tumor progression but tumor resistance to treatments is still a tough problem.

Ferroptosis is a novel form of programmed cell death along with iron accumulation, lipid hydro-peroxidation, and the change of mitochondria (3). Ferroptosis can be triggered by regulators like GPX4, system Xc^−^, and P53. GPX4 ensures the integrity of cell membrane by converting glutathione into an oxidized form and reducing lipid peroxides (3). System Xc^−^, composed of SLC7A11 and SLC3A2, is responsible for intaking the synthetic material of glutathione, and ferroptosis can be inhibited by suppressing system Xc^−^ (4). P53 inhibits SLC7A11 and blocks the absorption of cysteine to inhibit ferroptosis activation (5). Therefore, the regulation of ferroptosis is complicated and refers to multiple regulators.

A previous study reported that ferroptosis regulators like GPX4 are associated with tumor progression and tumor sensitivity to treatments, such as hepatocellular carcinoma, renal cell carcinoma, breast cancer, prostate cancer, and bladder carcinoma (6–8). Apoptosis-inducing factor mitochondria-associated 2 can inhibit GPX4 deletion induced ferroptosis, and pharmacological targeting of FSP1 strongly synergizes with GPX4 inhibitors to trigger ferroptosis (9). Decreased ACSL6 expression is associated with worse survival outcome in acute myelogenous leukemia (10). The expression profile of TRFC, FTH1, and FTL is positively correlated with tumor pathological grade and affects tumor progression like renal cell carcinoma, head and neck squamous cell carcinoma, and breast cancer (7, 11). Moreover, tumor metastasis is associated with low expression levels of MAP1LC3A in colorectal cancer (12). Therefore, ferroptosis related genes are tightly connected with gliomas progression.

In this study, a prognostic scoring system based on ferroptosis related genes expression is constructed which can predict GBM patient’s clinical outcome according to the TCGA, CGGA, GEO database, and our own samples. Moreover, high scoring samples also associate with aggressive subtype of GBM, mesenchymal, and classical, by performing RNA velocity in single cell RNA seq analysis. Critically, high scoring GBM cells communicate with macrophages, dendritic cell, naïve T cell, and microglial more active relative to low scoring GBM cells. In general, a ferroptosis activation scoring system is proposed and it can be applied to evaluate the aggressiveness of GBM. Ligand–receptor pairs are also proposed based on this system which may assist in revealing novel relationship of tumor cells and immunocytes.

## Materials and Methods

### Data Preparation

mRNA sequence data of gliomas are download from TCGA (https://xenabrowser.net/). Samples from CGGA (http://www.cgga.org.cn/) and GEO (https://www.ncbi.nlm.nih.gov/geo/) database are set as validation cohort. There are 137 GBM and 508 LGG samples in the training cohort, 84 GBM and 142 LGG samples from the CGGA sequencing data (CGGA1), 108 GBM and 155 LGG samples from the CGGA microarray (CGGA2), and 124 GBM and 170 LGG samples from GEO dataset (GSE108474). The Verhaak subtype of GBM is predicted as previous work (13).

GBM samples from Xiangya cohort were collected as previously study state (14).

Data for single-cell seq RNA analysis are downloaded from the GEO database (GSE84465). Expression data is normalized with R packages ‘Seurat’ and ‘NormalizeData’. Top 5,000 highly variable genes are identified with R package ‘FingVariableGenes’. Neoplastic, OPC, and other cells are offered previously, and immunocytes are classified with R package ‘scCATCH’. The distribution of cells components is mapped with R package ‘UMAP’. Subtypes of neoplastic cells in the single-cell RNAseq analysis are reproduced as previous work (15).

### Ferroptosis Activation Scoring Model

Forty-three ferroptosis related gene are selected according to previous research (16). Samples from the TCGA dataset are assigned to cluster1 or cluster2 based on ferroptosis related genes expression by performing the consensus clustering analysis.

Next, differential expression genes (DEGs) between cluster1 and cluster2 are identified with R package ‘limma’. The univariate Cox regression analysis and the elastic net regression analysis are employed to identify survival outcome associated genes. Ferroptosis activation score (FeAS) is calculated based on the principal components analysis:


$$\text{Ferroptosis activation score}=\text{Gen}{\text{e}}_{\text{HR }>1}*(\text{PC}1+\text{PC}2)-\text{Gen}{\text{e}}_{\text{HR}<1}*(\text{PC}1+\text{PC}2)$$

**Figure 1:**
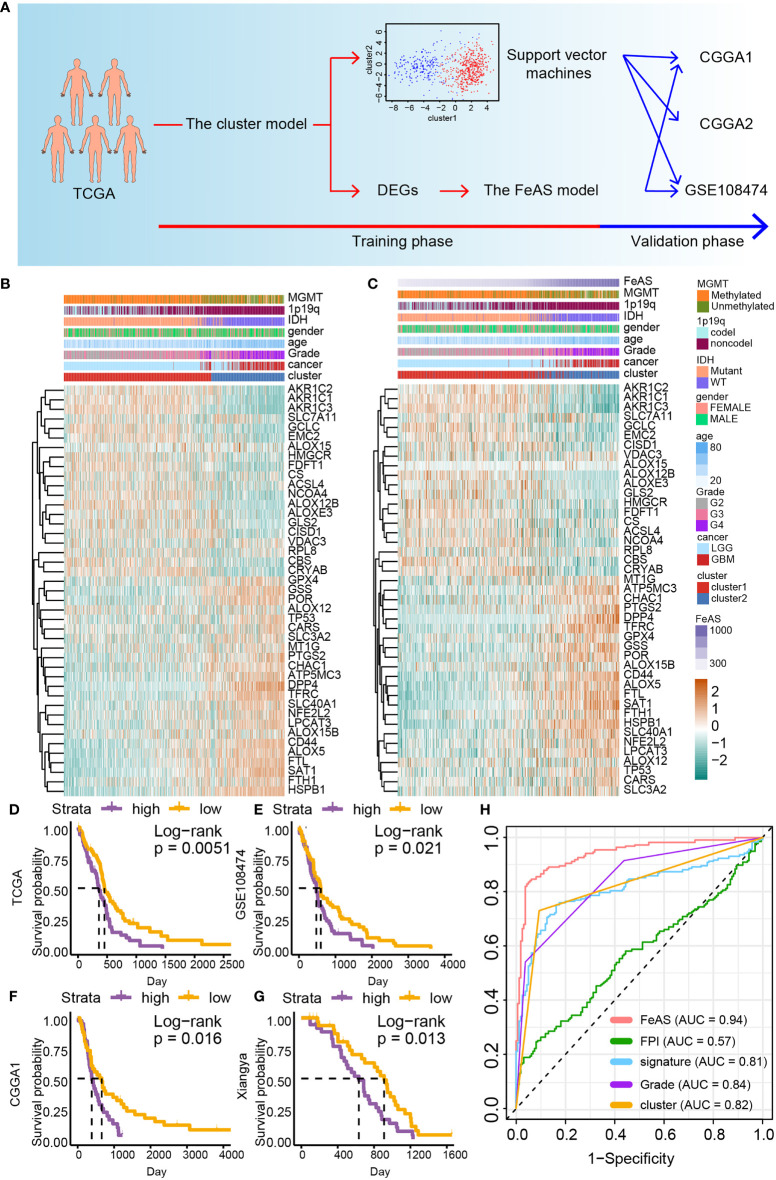
The construction of the FeAS model. **(A)** Flow chart shows the construction of the FeAS model. **(B)** Ferroptosis related gene expression and corresponding clinical feature based on the clustering model was illustrated with heatmap. **(C)** Ferroptosis related gene expression and corresponding clinical feature based on the FeAS model was illustrated with heatmap. Survival analysis based on the FeAS model in the GBM cohort in TCGA database (**D**, P value = 0.0051), GSE108474 database (**E**, P value = 0.021), CGGA1 database (**F**, P value = 0.016) and the Xiangya cohort (**G**, P value = 0.013). **(H)** Prognostic efficiency ability comparison between the FeAS model and other three ferroptosis models by introducing ROC curve.

The characteristics of cluster1 and cluster2 are learned with the support vector machine algorithm by R package ‘e1071’. Its sensitivity and specificity are evaluated with R package ‘caret’. Then, samples from CGGA1, CGGA2, and GSE108474 are grouped into cluster1 or cluster2. The FeAS model in the validation cohort is reproduced with a similar formula. The construction of the cluster model and the FeAS model is showed with schematic diagram (**Figure 1A**).

## Overall Survival Outcome Prediction

Samples are grouped into high or low FeAS group according to the median value of FeAS. Overall survival difference between high and low FeAS group is predicted with the Kaplan–Meier algorithm. The receiver operation characteristic (ROC) curve and area under curve (AUC) are generated to compare prognostic ability within different models.

### Biofunction Prediction

The GO/KEGG enrichment analysis based on the GSVA algorithm is performed with data from bulk RNA-seq analysis. Classic GO/KEGG analysis is performed in single cell RNA-seq analysis. The GSEA enrichment analysis is conducted based on DEGs between high and low FeAS group in the bulk RNA-seq analysis and single cell RNA-seq analysis.

### GBM Immune Landscape

The ESTIMATE algorithm is employed to depict the infiltration ratio of immunocytes, stromal cells, and gliomas cells. Then, tumor microenvironment cells components are analyzed by introducing the CIBERSORT algorithm (17) and the xCell algorithm (18) as previously reported.

Previous work proposed six immune subsets (Wound healing, IFN-γ Dominant, Immunologically Quiet, Inflammatory, Lymphocyte Depleted. and TGF-β Dominant) (19). The same classification is reproduced in this work using R package ‘ImmuneSubtypeClassifier’.

### RNA Velocity and Cells Communication

RNA velocity of tumor cells is calculated by package ‘velocity’ and ‘scVelo’ with python. Different state of GBM cells is mapped to show their internal transformation. Cross-talk between immunocytes and GBM cells is analyzed by R package ‘celltalker’, and differential ligand–receptor pairs are identified.

### Transcription Factor Regulatory Network Construction and Cells Communication

RcisTarget database of human is downloaded from https://resources.aertslab.org/cistarget/ for transcription factor regulatory network construction. R package ‘SCENIC’ is introduced to construct the network (20). AUCell algorithm is applied to evaluate transcription factor activation, and regulon modules are identified according to connection specificity index.

### Potential Sensitive Drug Prediction

The drug sensitive information and corresponding expression are obtained from PRISM Repurposing dataset (referred as ‘PRISM’ in the following text, https://depmap.org/portal/prism/) and Cancer Therapeutics Response Portal (referred as ‘CTRP v1’ and ‘CTRP v2’ in the following text, https://portals.broadinstitute.org/ctrp). Cells sensitivity to drugs is qualified as AUC value, and lower AUC value suggests higher sensitivity to potential drugs. The AUC of each sample in this study is calculated with R package ‘pRRophetic’ as previous work depicted (21).

Similar strategy was applied to data that downloaded from the CellMiner database (22). Approximately 50% growth-inhibitory level (GI50) is introduced to evaluate drug sensitivity, and lower GI50 represented higher drug sensitivity.

### Statistical Analysis

The Wilcox rank sum test is conducted to examine the difference between two comparisons while ANOVA test is employed for multiple comparisons. Fisher’s precision probability test is used for R*C contingency table which contained samples of <5. The Spearman correlation is introduced to evaluate relationship between metric variable. Log-rank test is performed for the overall survival analysis. Wilcox rank sum test and spearman correlation are performed during potential sensitive drugs selection. All analyses are performed by R (version 3.6.2) or python.

## Results

### Ferroptosis Activation Associates With Gliomas’ Subtypes and Predicts Gliomas Patient’s Survival Outcome

Samples in the TCGA dataset are clustered into two groups, cluster1 and cluster2, by performing the consensus clustering analysis (**Supplementary Figure 1A**). The support vector machine algorithm is employed to reproduce the clustering model in the validation cohort. Then, heatmap reveals the connection between the clustering model, gliomas clinical features and ferroptosis related gene expression. High grade gliomas, namely, GBM, IDH wild type gliomas, and MGMT unmethylated gliomas are related to samples in cluster2. Critically, the expression of ferroptosis resistance related gene (such as *GPX4*, *TFRC*, *FTH1*, and *FTL*) is up-regulated while ferroptosis sensitive related gene (like *AOLX12B*, *ACSL4*, and *AKRs*) is decreased in cluster2 than cluster1 suggesting that samples in cluster2 may resistant to ferroptosis (**Figure 1B**). Similar results are also verified in the validation datasets (**Supplementary Figures 1B–D**). Therefore, samples in cluster2 may be resistant to ferroptosis.

Then, the prognostic ability of the clustering model is examined with the overall survival analysis (**Supplementary Figure 2**). Samples from cluster1 manifest better survival outcome than cluster2 in the LGGGBM cohort (p <0.0001) and the LGG (p <0.0001) cohort. However, no significant survival outcome difference is noticed in the GBM cohort (p = 0.079). In the validation cohort, significant survival outcome difference is observed in the LGGGBM cohort (CGGA1: p <0.0001; CGGA2: p = 0.0054; GSE108474: p <0.0001) and the LGG cohort (CGGA1: p <0.0001; CGGA2: p = 0.0055; GSE108474: p <0.0001) but not in the GBM cohort (CGGA1: p = 0.12; CGGA2: p = 0.69; GSE108474: p = 0.29). Therefore, the clustering model indicates that the activation of ferroptosis is different between LGG and GBM, and ferroptosis sensitive samples exhibit longer survival time tendency.

### The FeAS Model Exhibits Great Prognostic Prediction Ability

In order to improve the accuracy of prognosis prediction ability of the clustering model, the FeAS model is further constructed with DEGs between cluster1 and cluster2 (**Supplementary Figure 3A**). The elastic net regression analysis is introduced to identify main contributors of the FeAS model (**Supplementary Figures 3B, C**) and FeAS of each sample is calculated. The distribution of FeAS of samples and corresponding clinical features, ferroptosis related gene expression is introduced by heatmap in the TCGA dataset (**Figure 1C**) and validation cohort (**Supplementary Figures 3D, E**). Ferroptosis resistance related gene are also preferential expressed in high FeAS GBM samples. High FeAS GBM samples are also associated with malignancy clinical features (like wildtype IDH gliomas, non-codel 1p19q gliomas, and unmethylated MGMT gliomas).

Overall survival analysis suggests that high FeAS samples show shorter median overall survival time than low FeAS samples in the LGGGBM and LGG cohort (**Supplementary Figure 4**). Critically, high FeAS samples show worse survival outcome than low FeAS samples in the GBM cohort from the TCGA (P value = 0.01; **Figure 1D**), GSE108474 (P value = 0.021; **Figure 1E**), CGGA1 (P value = 0.0098; **Figure 1F**) and Xiangya cohort (P value = 0.0013; **Figure 1G**).

Comparing with previous public prognostic models based on ferroptosis related gene, the FeAS model (AUC: 0.94) shows the highest accuracy in predicting patient’s survival outcome than other three models [FPI from work of Z. Liu et al., and the AUC value is 0.57 (8); signature from work of H. Liu et al., and the AUC value is 0.81 (23)]. Moreover, the AUC value of the FeAS model is also higher that the clustering model (AUC: 0.82) and gliomas pathological grades (AUC: 0.84) (**Figure 1H**). Taken together, the FeAS model exhibits the highest accuracy in predicting gliomas’ clinical outcome than other three models, and it can be applied to predict GBM prognosis. Therefore, we focus on exploring the role of ferroptosis in GBM based on the FeAS model.

### High FeAS Glioblastoma Are More Aggressive Than Low FeAS Glioblastoma

**Figure 2:**
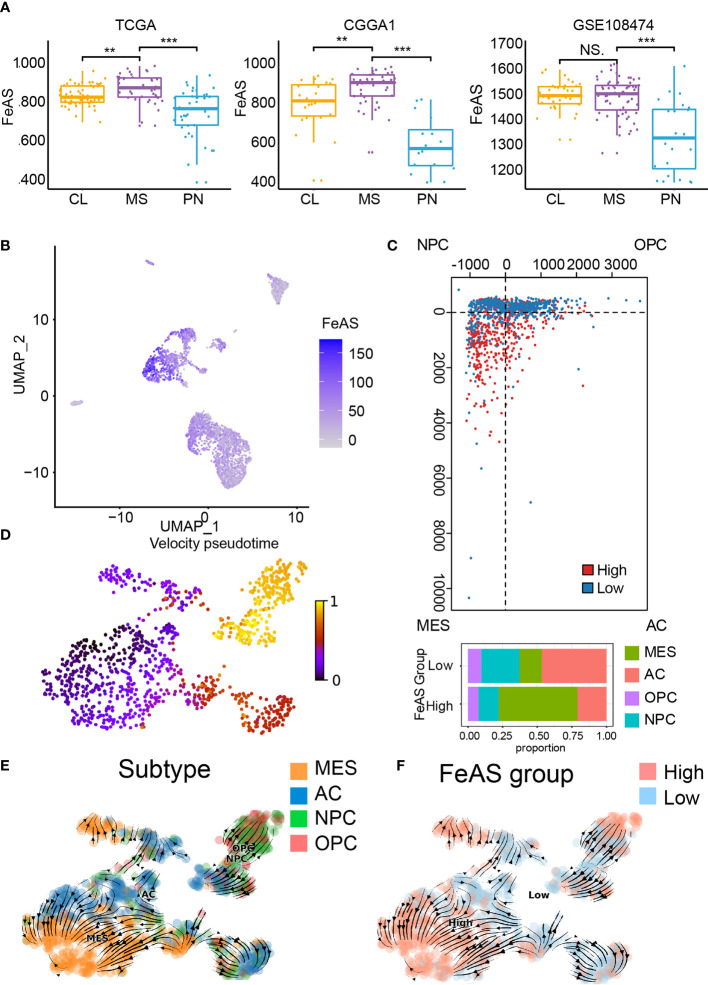
Association between ferroptosis and GBM aggressiveness. **(A)** Distribution of FeAS in GBM subtypes according to bulk RNA-seq analysis in the TCGA, CGGA1, and GSE108474 database. **(B)** Distribution of FeAS in single cell RNA-seq analysis. **(C)** The subtype of GBM cells in the FeAS model. **(D)** RNA velocity illustrated by pseudo-time analysis indicating GBM cells aggressiveness difference. **(E)** Integration of RNA velocity and the subtype of GBM cells. **(F)** Integration of RNA velocity and FeAS of GBM cells. CL, classical; MES, mesenchymal; PN, proneural. NS, no significant; **P < 0.01; ***P < 0.001.

Next, we investigated the relationship between FeAS and the Verhaak GBM subtype. Previous work reported that mesenchymal and classical are two aggressive GBM subtypes than proneural. In this work, we notice that high FeAS GBM are more likely to be defined as mesenchymal or classical GBM in both training and validation cohort (**Figure 2A**). Another study based on single cell RNA-seq analysis revealed the tumor cells genomic characteristic in different GBM subtype, and mesenchymal GBM and MES-like cells shared similar genomic characteristic (15). Therefore, we explored the association between FeAS and GBM cells’ genomic characteristic by introducing single cell RNA-seq analysis.

Cells’ identity is identified by their biomarkers and the distribution of those cells is mapped by R package ‘UMAP’ (**Supplementary Figures 5A, B**). FeAS of each cell is calculated according to the formula (**Supplementary Figure 5C**), and tumor cells show higher FeAS than other cells implying dysregulated ferroptosis in tumor cells (**Figure 2B**).

The relationship between GBM cells’ genomic character and FeAS suggested that high FeAS group contains more MES-like cells while other subtype cells enrich in low FeAS group (**Figure 2C**). RNA velocity was calculated in previous study to evaluate the abundance of unspliced and spliced RNA in cells which can reflect cell evolution pathway. The RNA velocity of GBM cells is calculated and a clear evolution pathway is mapped (**Figure 2D** and **Supplementary Figure 5D**). MES-like cells mostly locate at the end of the differentiation pathway while OPC-like and NPC-like cells are enriched at the apex (**Figure 2E**). Critically, a lineage from low FeAS GBM cells to high FeAS GBM cells is also traced (**Figure 2F**), and this lineage is similar with the differentiation pathway of GBM cells’ subtype. Taken together, high FeAS GBM cells represent more aggressive GBM cells subtype than low FeAS GBM cells. Together, ferroptosis is dysregulated in tumor cells and its activation highly associates with the subtype of GBM cells.

### Transcription Factor Differentially Activated in High and Low FeAS Glioblastoma

**Figure 3:**
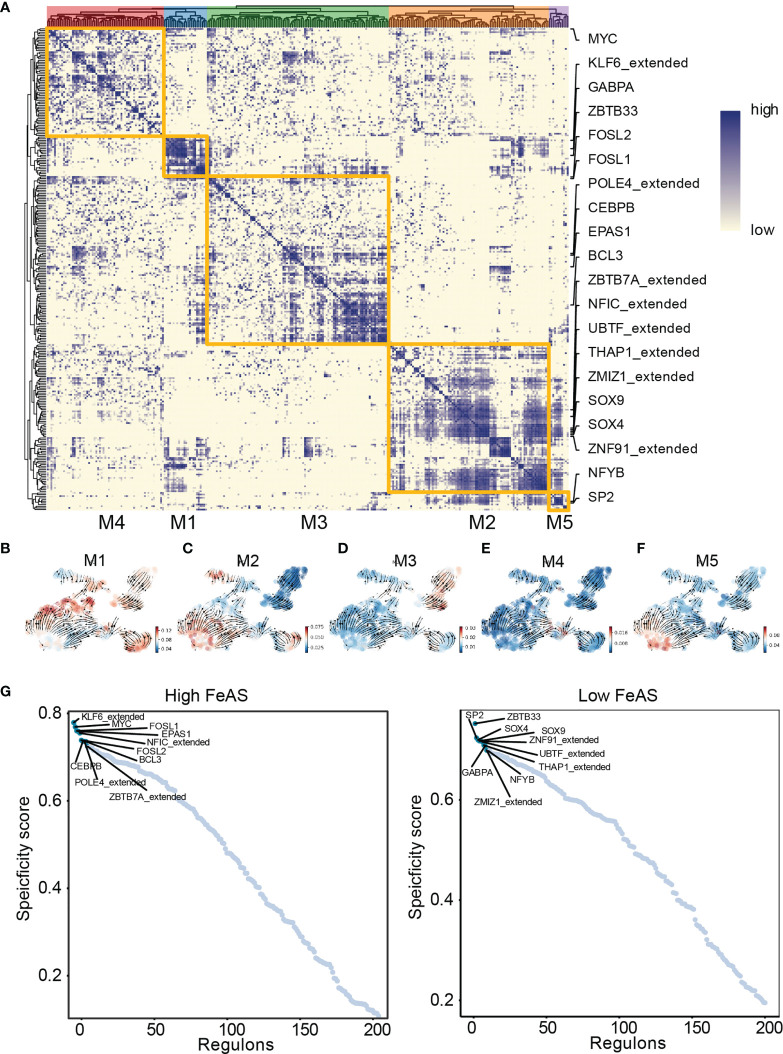
Transcription factor activation difference between high and low FeAS GBM cells. **(A)** GBM cells can be grouped in five modules according to the cooperation of different transcription factors. **(B–F)** Potential relationship between the scoring system and those modules based on RNA velocity. **(G)** Top 10 differential activated transcription factor in high and low FeAS samples respectively.

Next, we look into the activation of transcription factor in high and low FeAS GBM cells (**Figure 3A**). Considering transcription factors regulate certain gene expression mutually, we cluster different modules (M1, M2, M3, M4, M5) based on that. Each module represents a bunch of transcription factors which may cooperate with each other. Regulon activity scores (RAS) of each module is calculated based on Connection Specificity Index according to previous work (24). RNA velocity is also performed to map the relationship between those modules and the scoring system. It seems that high RAS of M1 exists in both high and low FeAS group (**Figure 3B**). In the meantime, RAS of M2 (**Figure 3C**) is positive correlated with FeAS, while RAS of M3 and M5 (**Figure 3F**) are negative correlated with FeAS (**Figure 3D**). There is no significant distribution difference in RAS of M4 between high and low FeAS group (**Figure 3E**).

Next, we select the top 10 transcription factors from high (KLF6_extended from M1, MYC from M4, FOSL1 from M1, EPAS1 from M3, NFIC_extended from M2, FOSL2 from M1, CEBPB from M3, POLE4_extended from M3, BCL3 from M3, and ZBTB7A_extended from M3) and low (ZBTB33 from M1, SP2 from M5, SOX4 from M2, SOX9 from M2, ZNF91_extended from M2, UBTF_extended from M2, THAP1_extended from M2, GABPA from M1, ZMIZ1_extended from M2, and NFYB from M5) FeAS group for further analysis (**Figure 3G**). Their expression is about mapped in high and low FeAS group (**Supplementary Figure 6**).

Thus, we perform GO and KEGG enrichment analysis based on those transcription factors. In high FeAS GBM cells, we selected MYC (**Supplementary Figure 7A**), KLF6_extend (**Supplementary Figure 7B**), FOSL1 (**Supplementary Figure 7C**) and FOSL2 (**Supplementary Figure 7D**) as a representative. Pathways related to cell adhesion like extracellular matrix binding, cell adhesion molecule binding, cadherin binding involved in cell-cell adhesion, extracellular matrix structural constituent, collagen binding, ECM-receptor interaction, and focal adhesion were preferentially activated in high FeAS GBM cells which may explain their aggressiveness.

On the other hand, SP2 (**Supplementary Figure 8A**), ZBTB33 (**Supplementary Figure 8B**), ZNF91_extended (**Supplementary Figure 8C**), SOX4 (**Supplementary Figure 8D**), and SOX9 (**Supplementary Figure 8E**) are marked as biomarkers of low FeAS GBM cells, and higher enrichment of pathways like DNA replication, ubiquitination modification is identified. For instance, DNA replication origin binding, cell cycle, ubiquitin protein ligase binding, histone binding, DNA-binding transcription repressor activity, RNA transport, mRNA surveillance pathway, protein phosphatase 1 binding, and AMPK signaling pathway are enriched in those cells.

### Immune Related Pathways Selectively Activate in High FeAS Glioblastoma

In the cluster model, GO and KEGG enrichment analysis based on GSEA analysis suggested that immunocytes related pathways, like IL6 associated pathway, macrophage related pathways, JAK-STAT signaling pathway, and TNF signaling pathway are preferentially activated in cluster2 samples than cluster1 samples (**Supplementary Figures 9A, B**). Results suggested immunogencity is different between cluster1 and cluster2 samples. Therefore, we further explored those results in the FeAS scoring system.

**Figure 4:**
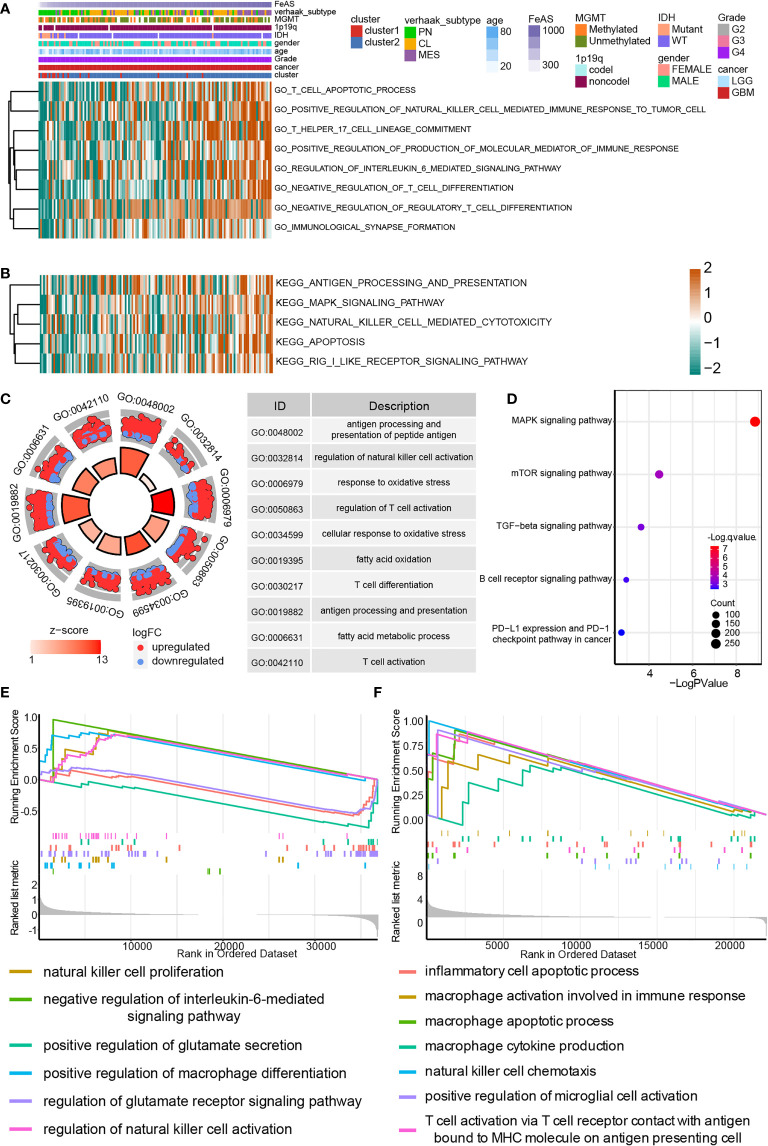
Biofunction analysis based on bulk RNA-seq analysis and single cell RNA-seq analysis in GBM. **(A)** GO enrichment analysis based on the GSVA algorithm in bulk RNA-seq analysis. **(B)** KEGG enrichment analysis based on the GSVA algorithm in bulk RNA-seq analysis. **(C)** GO enrichment analysis based on differential expression genes between high and low FeAS samples in single cell RNA-seq analysis. **(D)** KEGG enrichment analysis based on differential expression genes between high and low FeAS samples in single cell RNA-seq analysis. GSEA enrichment analysis based on bulk RNA-seq analysis **(E)** and single cell RNA-seq analysis **(F)**.

The GO enrichment analysis based on the GSVA analysis on GBM samples from TCGA database suggests that high FeAS GBM samples are associated with the activation of immune related pathways (**Figure 4A**). For instance, positive regulation of production of molecular mediator of immune response, T cell related pathways, regulation of interleukin 6 mediated signaling pathway, immunological synapse formation, and positive regulation of natural killer cell mediated immune response to tumor cell. In the meantime, MAPK signaling pathway, antigen processing and presentation, natural killer cell mediated cytotoxicity, apoptosis and RIG I like receptor signaling pathway are activated in high FeAS GBM samples according to the KEGG enrichment analysis (**Figure 4B**). By verifying the GO/KEGG enrichment analysis with the single cell RNA-seq analysis, similar conclusion can be obtained. Activation of antigen processing and presentation of peptide antigen, regulation of natural killer cell activation, response to oxidative stress, T cell related pathways, fatty acid metabolic process are higher in high FeAS GBM cells according to the GO enrichment analysis (**Figure 4C**); while pathways like MAPK signaling pathway, mTOR signaling pathway, TGF-β signaling pathway, B cell receptor signaling pathway, PD-L1 expression, and PD-1 checkpoint pathway in cancer are activated in high FeAS GBM cells by conducting the KEGG enrichment analysis (**Figure 4D**).

The GSEA analysis based on bulk RNA-seq analysis and single cell RNA-seq analysis are also employed (**Figures 4E, F**). Results from bulk RNA-seq analysis suggests that pathways like natural killer cell proliferation, negative regulation of interleukin-6 mediated signaling pathway, positive regulation of macrophage differentiation in high FeAS GBM samples; and pathways like positive regulation of glutamate secretion, regulation of glutamate receptor signaling pathway are activated in low FeAS GBM samples. In the single cell RNA-seq analysis, inflammatory cell apoptotic process, macrophage related pathways, natural killer cell chemotaxis, microglial cell and T cell activation are higher enriched in high FeAS GBM cells. Therefore, the immune landscape may different between high and low FeAS GBM samples, and which may also contribute to the variety clinical outcome.

### FeAS of Glioblastoma Influence Macrophage, Dendritic Cells, NK Cells and T Cells Enrichment

Next, we explore the connection between tumor immune landscape and the scoring system according to an immune subtype which is proposed by previous work (19). GBM samples mostly consist of Lymphocytes Depleted subtype while Lymphocytes Depleted subtype and Immunogenetic Quiet subtype were dominate subtypes in LGG samples. Similar composition can also be noticed in our work (**Supplementary Tables 1**, **2** and **Supplementary Figure 10**). It also suggested that Lymphocytes Depleted subtype manifested worse survival outcome relative to Immunologically Quiet subtype. Similarly, low scoring samples is associated with Immunologically Quiet subtype, and shows better prognosis comparing with high scoring samples in TCGA (**Supplementary Figure 10A**), CGGA1 (**Supplementary Figure 10B**), and GSE108474 database (**Supplementary Figure 10C**).

But what interesting is that the proportion of Lymphocytes Depleted subtype in low scoring samples sharply increases to the same level as high scoring samples when only analyze GBM samples (**Supplementary Table 2** and **Supplementary Figures 10D–F**) implying another unveiled mechanism which may contribute to their prognosis difference. In the meantime, Immunogenicity Quiet subtype nearly vanished in all GBM samples. Since Lymphocyte Depleted subtype was labeled as samples with prominent macrophage signature, Th1 suppressed and high M2 macrophage response, it is vital to analyze the immunocytes infiltration difference.

**Figure 5:**
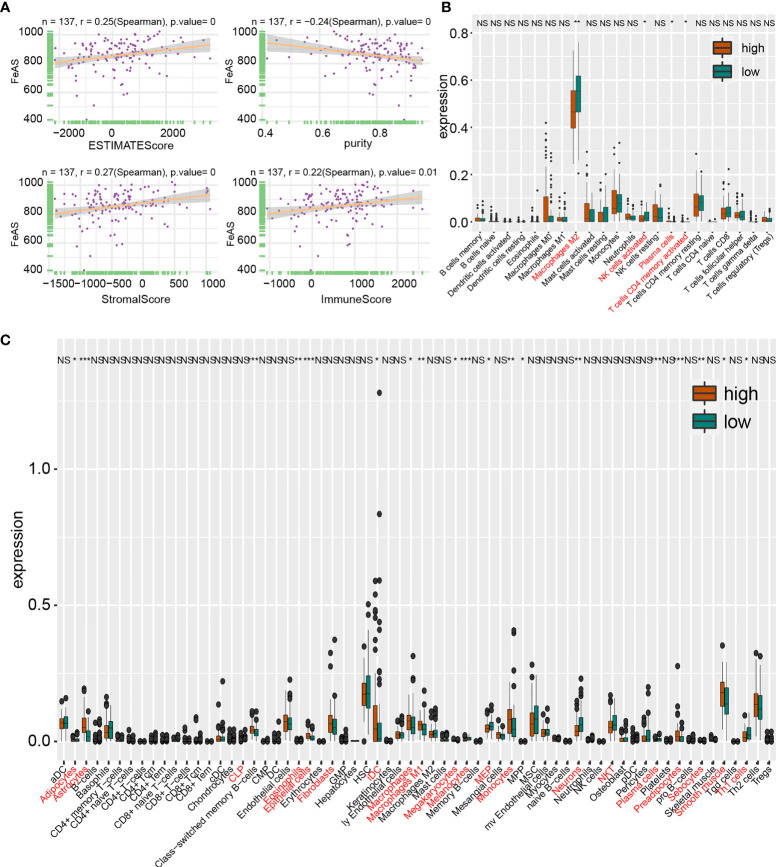
Tumor immune landscape based on bulk RNA-seq analysis in TCGA database. **(A)** Correlation of ESTIMATE score, stromal score, immune score and tumor purity with FeAS. Immunocytes infiltration ratio in high and low FeAS samples according to CIBERSORT algorithm **(B)** and xCell algorithm **(C)**. NS, no significant. *P < 0.05; **P < 0.01; ***P < 0.001.

First, ESTIMATE algorithm is introduced to offer an overview of immune landscape of GBM samples. The map of immune landscape shows that high FeAS positively correlates with ESTIMATE score, immune score and stromal score; and negatively correlates with tumor purity in the GBM cohort from TCGA (**Figure 5A**), CGGA1 database, and Xiangya (**Supplementary Figures 11A, B**). Thus, more immunocytes and stromal cells are infiltrated in high FeAS GBM samples.

Then, CIBERSORT algorithm and xCell algorithm are conducted on GBM samples to qualify immunocytes infiltration. The former algorithm suggests that higher M2 macrophage, activated memory CD4+ T cells and activated NK cells in low FeAS GBM samples in TCGA database (**Figure 5B**); more M0 macrophage and resting memory CD4+ T cells in high FeAS GBM samples in CGGA1 database (**Supplementary Figure 11C**); and M0 macrophage and plasma cells in high FeAS samples in Xiangya cohort. As for low FeAS GBM cells, high filtration ratio of monocytes are noticed in low FeAS samples from Xiangya cohort (**Supplementary Figure 11D**). Similar component can also be noticed in analysis based on the cluster model (**Supplementary Figures 9C–H**).

The latter algorithm shows that higher infiltration ratio of M1 macrophage and immature dendritic cell (iDC) in high FeAS samples; and natural killer cells and Th1 cells are enriched in low FeAS samples in TCGA database (**Figure 5C**). Similar conclusion can also be obtained in the validation cohort (**Supplementary Figures 11E, F**). Even if most of samples, no matter they come from high or low FeAS group, are categorized as Lymphocytes Depleted subtype, diversity on macrophage signature is still existed. For instance, high and low FeAS GBM share similar M2 macrophage infiltration ratio, but higher M1 macrophage and higher Th1 cells are found in high and low FeAS GBM samples respectively. Lower Th1 cells in high FeAS GBM imply much severe immunosuppressive microenvironment. Taken together, infiltration of macrophage, T cells, dendritic cells and NK cells in tumor microenvironment is altered according to FeAS. More importantly, the microenvironment of high FeAS GBM samples possessed is suppressed more than low FeAS samples.

### Novel Ligand–Receptors Pairs Between Immunocytes and High FeAS Glioblastoma Cells

**Figure 6:**
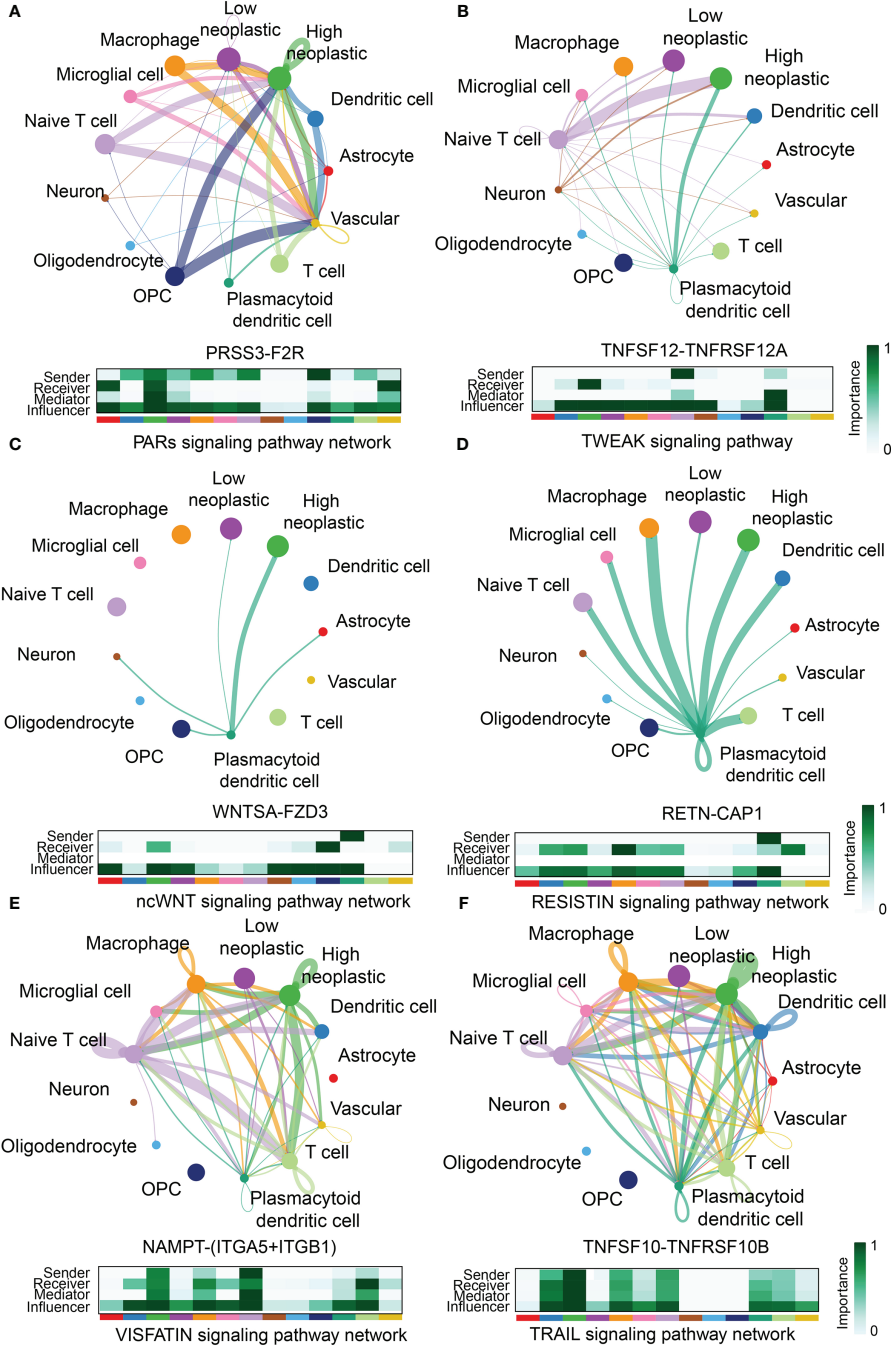
Novel ligand–receptor pairs difference between high and low FeAS samples. **(A)** High FeAS cells communicate with macrophage, naïve T cell, and dendritic cells through PRSS3-F2R. **(B)** High FeAS cells communicate with naïve T cell and plasmacytoid dendritic cell through TNFSF12-TNFSF12A. **(C)** High FeAS cells communicate with plasmacytoid dendritic cell through WNTSA-FZD3. **(D)** High FeAS cells communicate with plasmacytoid dendritic cell through RETN-CAP1. **(E)** High FeAS cells communicate with macrophage, naïve T cell and T cells through NAMPT-(ITGA5+ITGB1). **(F)** High FeAS cells communicate with macrophage, microglial cell, naïve T cell, T cells and plasmacytoid dendritic cell through TNFSF10-TNFRSF10B.

Although most GBM samples are grouped as Lymphocytes Depleted subtype, immunocytes infiltration ratio still manifest slightly difference between high and low FeAS GBM samples. Therefore, we investigated the communications between immunocytes and GBM cells with the single cell RNA-seq analysis. As illustrated, high FeAS GBM cells communicate with macrophage, naïve T cell, microglial cell, and dendritic cell actively. Different roles of cells act in cells communication are introduced in previous research (25, 26). In our work, high FeAS cells can receive signal from naïve T cell, macrophage, dendritic cell through PARs signaling pathway (PRSS3-F2R, **Figure 6A**), TWEAK signaling pathway (TNFSF12-TNFRSF12A, **Figure 6B**), ncWNT signaling pathway (WNTSA-FZD3, **Figure 6C**), RESISTIN signaling pathway (RETN-CAP1, **Figure 6D**), VISFATIN signaling pathway (NAMPT-(ITGA5+ITGB1), **Figure 6E**), TRAIL signaling pathway (TNFSF10-TNFRSF10B, **Figure 6F**), SPP1 signaling pathway (SPP1-CD44, **Supplementary Figure 12A**) and VEGF signaling pathway (VEGFB-VEGFR1, **Supplementary Figure 12B**). Additionally, high FeAS GBM cells can also send signal to those cells by PROS signaling pathway (PROS1-AXL, **Supplementary Figure 12C**), LT signaling pathway (LTA-TNFRSF1B, **Supplementary Figure 12D**), ANNEXIN signaling pathway (ANXA1-FPR1, **Supplementary Figure 12E**) and MIF signaling pathway (MIF-(CD74+CXCR4), **Supplementary Figure 12F**). In summary, high FeAS GBM cells communicate with macrophage, microglial, naïve T cell and dendritic cell more active by comparing with low FeAS GBM cells which may explain the immune landscape difference.

### Potential Targeted Drugs for High FeAS Glioblastoma Cells

**Figure 7:**
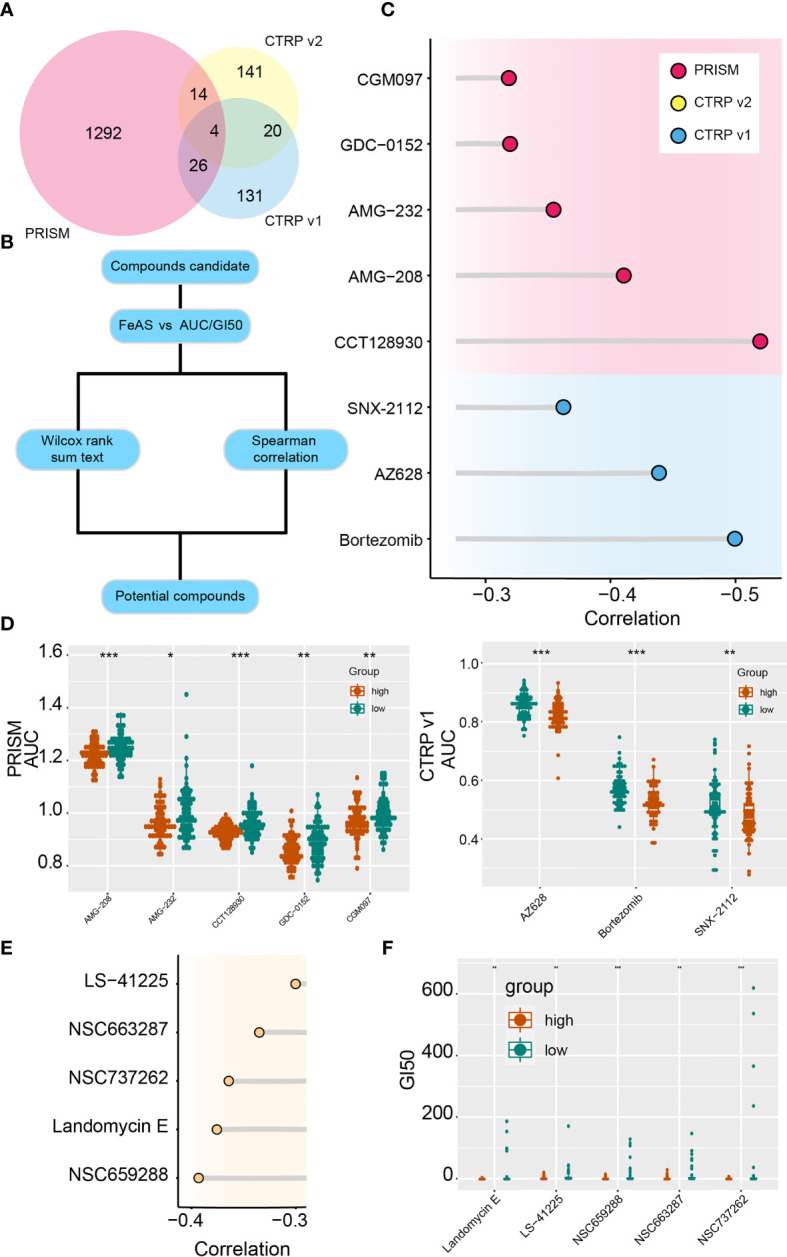
Potential targeted drugs according to the FeAS model. **(A)** Venn chart shows the number of drugs in PRISM dataset and two CTRP database. **(B)** Flow chart illustrates the potential compounds based on the FeAS model. **(C)** Correlation between the AUC value of potential drugs and the FeAS of each sample. **(D)** Distribution of the AUC value of potential drugs in the FeAS model. **(E)** Correlation between GI50 and FeAS. **(F)** The distribution of GI50 of each compound based on the FeAS model. *P < 0.05; **P < 0.01; ***P < 0.001.

Potential sensitive drugs are predicted base on the expression data and drug sensitive data from the PRISM and CTRP database, and overlapped drugs are filtered out (**Figure 7A**). Lower AUC value represents higher sensitivity to drugs. Top differential AUC value between high and low FeAS GBM samples, and Spearman’s correlation with FeAS >0.3 are set as threshold for compounds selection (**Figure 7B**). Spearman correlation is introduced in **Figure 7C**, and the distribution of each drug’s AUC is mapped in **Figure 6D**. CGM097, AMG-232, AMG-208, GDC-0152, and CCT128930 are identified from the PRISM database; SNX-2112, AZ628, and bortezomib are filtered out from CTRP v1 database. No potential compounds are found in CTRP v2 database.

Moreover, drug sensitivity from the CellMiner database is also predicted base on the FeAS model with similar strategy. NSC663287, NSC737262, NSC659288, landomycin E, and LS-41225 are identified as high FeAS samples sensitive drugs (**Figures 7E, F**). Therefore, low FeAS samples are supposed to sensitive to those compounds, and those compounds may novel options for future GBM treatments.

## Discussion

Ferroptosis is recognized as programmed cell death and characterized with features like lipid hydro-peroxidation, iron accumulation. Recent studies proposed that ferroptosis is widely involved in tumor progression and responsible for tumor resistance to chemo- and radio-therapy (27, 28). For instance, activated ferroptosis inhibited head and neck carcinoma (29) and triple-negative breast cancer progression (30). In our work, high sensitivity prognostic model, the FeAS model, is proposed based on ferroptosis related genes expression. This model shows higher accuracy in predicting gliomas patient’s prognosis, especially in GBM, implying its wide application. Previous studies also reported that ferroptosis is associated with gliomas progression, growth and resistance to temozolomide, standard chemotherapeutic drug for gliomas (23, 31). Therefore, ferroptosis in gliomas, especially in GBM, progression still require more attention.

The Verhaak classification of GBM (including mesenchymal, classical, proneural, and neural) (3) and its updated version (neural GBM was discarded due to the contamination of normal brain tissue) (13) were proposed in recent years, and this classification can predict gliomas prognosis precisely. Mesenchymal GBM is viewed as the most aggressive subtype in the Verhaak classification than other subtypes. In our work, we discover that high FeAS GBM are more likely be defined as mesenchymal or classical subtype. To step further, single cell RNA-seq analysis subdivided GBM cells into four groups (MES-like, AC-like, OPC-like and NPC-like) (15). The mesenchymal GBM usually contains more MES-like cells. High FeAS GBM cells are tended to be grouped as MES-like cells after reproducing this classification. Therefore, the FeAS system can also evaluate the aggressiveness of GBM.

The transition of GBM subtype, the proneural–mesenchymal transition, during GBM progression is believed that involve in GBM recurrence and resistance to treatment. Following researches on this transition revealed that mesenchymal GBM showed more aggressive growth pattern and resistant to cancer treatment than other subtypes, and cells component also altered during this transition. We further confirm this cells component transition by integrating RNA velocity and single GBM cells classification. Critically, we also notice a pathway from low FeAS to high FeAS align to this transition. Since ferroptosis resistant related genes are up-regulated in high FeAS samples, it may be possible to reverse this transition by improving cells sensitivity to ferroptosis.

RAS of those modules suggest that M2, M3, and M5 are activated in high FeAS group, and M1 is activated in both high and low FeAS group according to their RNA velocity. As for M4, there is no significant difference between high and low FeAS group. Together, those results suggest that M3 and M5 may be activated in aggressive subtypes, M1 and M4 are activated in both aggressive and non-aggressive subtypes.

However, the role M2 is more complicated. According to the top 10 transcription factor from high and low FeAS group, more transcription factors from M2 are identified in low FeAS group than high FeAS group. In the meantime, several researches reported that SOX4 can inhibit GBM cells proliferation by inducing cells to exit cell cycle (32) while SOX9 promotes GBM progression (33). Therefore, it is complicated to assume that if M2 is only activated in aggressive/non-aggressive subtypes or mediates the subtype transition.

Immune subtype based on previous study is performed, and Lymphocyte Depleted subtype is identified as prominent type either in high or low FeAS GBM samples. The characteristics of this subtype are high macrophage signatures, high M2 macrophage and low Th1 cells infiltration, and worst prognosis comparing with other six subtypes according to previous research (19). Interestingly, high and low FeAS GBM samples still manifest significant prognosis difference in spite of their similar composition which implies an unrevealed mechanism. Considering that results from biofunction prediction suggests immune relate pathways are activated in high FeAS samples. Immunocytes infiltration is further analyzed. Macrophage, NK cells, Th1 cells and dendritic cells show differentially infiltration ratio between high and low FeAS GBM, which can also be verified in the validation cohort. Higher level of M0 and M1 macrophage is noticed in high FeAS GBM samples than low FeAS GBM samples but there is no difference on M2 macrophage. Correspondingly, the ratio of NK cells and dendritic cells also altered which may associate with GBM cells response to ferroptosis.

Then, we predict multiple potential ligand–receptor pairs between GBM cells and immunocytes and endeavor to explore their potential mechanism. Some of them have already been reported. For instance, the roles of PROS1-AXL (34), LAT-TNFRSF1B (35), ANXA1-FPR1 (36, 37), MIF-CD74 (38–40), SPP1-CD44 (41), VEGFB-VEGFR (42), IL6-IL6R (43), and OSM-OSMR (44) have been confirmed in macrophage activation in GBM. Additionally, more novel combinations are first proposed in this work, including TRAIL signaling pathway (TNFSF10-TNFRSF10B), TWEAK signaling pathway (TNFSF12-TNFRSF12A), VISFATIN signaling pathway (NAMPT-(ITGA5+OTGB1)), ncWNT signaling pathway (WNTSA-FZD3), PARs signaling pathway (PRSS3-F2R), and RESISTIN signaling pathway (RETN-CAP1). Their roles in mediating immune response and affecting immunocytes have been proved in other areas but their connection with GBM is elusive. The recent study reported that immunotherapy-activated CD8 positive T cells affected gliomas immunotherapeutic response by inducing ferroptosis (45). Moreover, triggering ferroptosis activation inhibited gliomas progression (46) and reversed gliomas resistance to temozolomide (47). Taken together, ferroptosis may bridge GBM and immunocytes infiltration to affect GBM progression.

Potential targeted drugs for high FeAS samples are predicted.CGM097 (48) and AMG-232 (49), inhibitor of MDM2, can bind to TP53 to affect GBM progression. Bortezomib improves GBM sensitivity to temozolomide (50) and NK cell cytotoxicity (51). CCT128930 can inhibit GBM cell line, U87MG, progression through inhibiting Akt2 (52) but further examination is required. AMG-208, inhibitor of c-MET, was once considered as novel aspects for treating GBM but no further updating (53). Moreover, two drugs, SNX2112 (54, 55) and AZ628 (56, 57), have been proved that can inhibit tumor progression through inducing apoptosis and MAPK signaling pathway. However, their association with GBM progression or ferroptosis are still unknown.

In this study, we established a scoring model based on ferroptosis related gene in glioblastoma samples. High FeAS samples show more aggressive growth pattern and worse clinical outcome than low FeAS samples. Tumor cells with different FeAS communicate with immunocytes is also distinct implying that ferroptosis activation may modulate immunocytes function. We assumed that by targeting to high FeAS samples may improve patient’s prognosis, and novel potential compounds was also predicted by performing machine learning algorithm. In summary, the FeAS model can evaluate glioblastoma aggressiveness, modulate cross-talk with immunocytes and offer suggestion to chemotherapy.

## Data Availability Statement

The original contributions presented in the study are included in the article/**Supplementary Material**. The original data has been uploaded to China National Center for Bioinformation (ID: HRA001618). Further inquiries can be directed to the corresponding authors.

## Ethics Statement

The studies involving human participants were reviewed and approved by the Xiangya Hospital. The patients/participants provided their written informed consent to participate in this study.

## Author Contributions

Manuscript preparation, data analysis: ZW and ZD. Assistants in data analysis: LZ and BX. Background investigation: HZ, FF, and LZ. Data collection: XZ. Project designation, Funding and Supervising: ZL, QC and KY. All authors contributed to the article and approved the submitted version.

## Funding

This work is supported by the National Nature Science Foundation of China (No. 82073893, 81873635,and 81703622); the China Postdoctoral Science Foundation (No. 2018M633002); the Natural Science Foundation of Hunan Province (Nos. 2018JJ3838, 2018SK2101, and 2019JJ50948); the Hunan Provincial Health and Health Committee Foundation of China (C2019186); Xiangya Hospital Central South University postdoctoral foundation; and the Fundamental Research Funds for the Central Universities of Central South University (No. 2021zzts1027).

## Conflict of Interest

The authors declare that the research was conducted in the absence of any commercial or financial relationships that could be construed as a potential conflict of interest.

## Publisher’s Note

All claims expressed in this article are solely those of the authors and do not necessarily represent those of their affiliated organizations, or those of the publisher, the editors and the reviewers. Any product that may be evaluated in this article, or claim that may be made by its manufacturer, is not guaranteed or endorsed by the publisher.
