# Supplementary material for: Ferroptosis Activation Scoring Model Assists in Chemotherapeutic Agents’ Selection and Mediates Cross-Talk With Immunocytes in Malignant Glioblastoma
Source: Front Immunol. 2022 Jan 19;12:747408. doi: 10.3389/fimmu.2021.747408 (PMC8807564; doi:10.3389/fimmu.2021.747408)
Supplement: Supplementary file 1 [file DataSheet_1.pdf]

Supplementary table 1 | Immune subtype based on the scoring system in gliomas from TCGA, CGGA1 and GSE108474 database.

	TCGA		CGGA1		GSE108474	
	high	low	high	low	high	low
Wound Healing	3	0	0	0	0	0
IFN-γ Dominant	0	0	0	0	6	2
Inflammatory	5	1	7	1	14	57
Lymphocyte Depleted	252	31	98	19	184	141
Immunologically Quiet	61	291	7	93	0	7
TGF-β Dominant	1	0	1	0	3	0

Supplementary table 2 | Immune subtype based on the scoring system in GBM from TCGA, CGGA1 and GSE108474 database.

	TCGA		CGGA1		GSE108474	
	high	low	high	low	high	low
Wound Healing	1	2	0	0	0	0
IFN-γ Dominant	0	0	0	0	1	1
Inflammatory	0	0	2	1	3	12
Lymphocyte Depleted	67	66	39	35	57	48
Immunologically Quiet	0	1	0	6	0	1
TGF-β Dominant	0	0	1	0	1	0

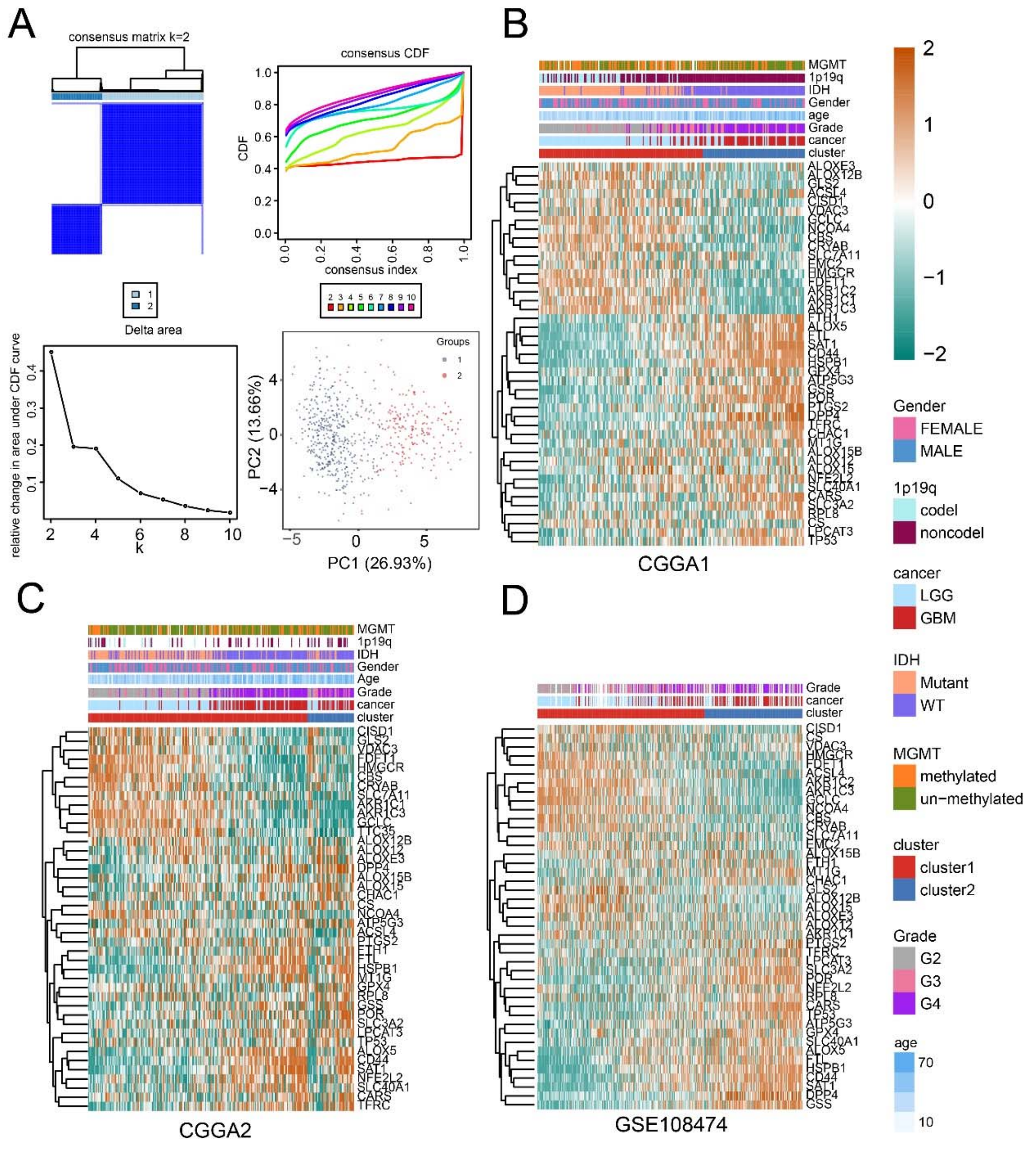

Supplementary Figure 1 | The construction of the clustering model. (A) Details about the construction of the clustering model. Ferroptosis related expression along with clinical features in the clustering model in the CGGA1 (B), CGGA2 (C) and GSE108474 database (D).

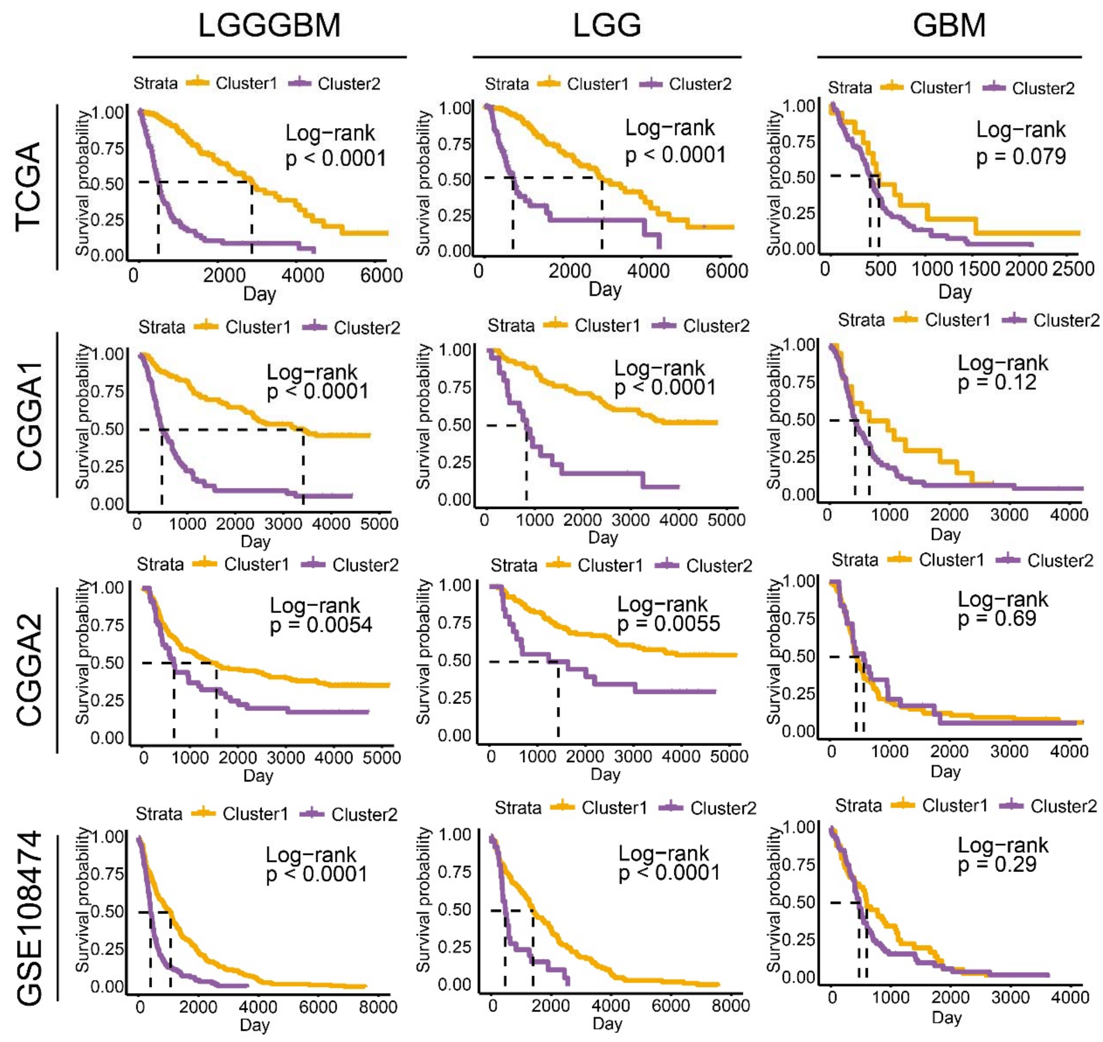

Supplementary Figure 2 | Overall survival analysis based on the clustering model in the TCGA, CGGA1, CGGA2 and GSE108474 database. Samples from cluster1 show worse survival outcome than cluster2 in the LGGGBM cohort (TCGA: P value < 0.0001; CGAA1: P value < 0.0001; CGGA2: P value < 0.0054; GSE108474: P value < 0.0001). Samples from cluster1 show worse survival outcome than cluster2 in the LGG cohort (TCGA: P value < 0.0001; CGAA1: P value < 0.0001; CGGA2: P value = 0.0055; GSE108474: P value < 0.0001). No significant survival outcome different is noticed between cluster1 show and cluster2 in the GBM cohort (TCGA: P value = 0.079; CGAA1: P value = 0.12; CGGA2: P value = 0.69; GSE108474: P value = 0.29).

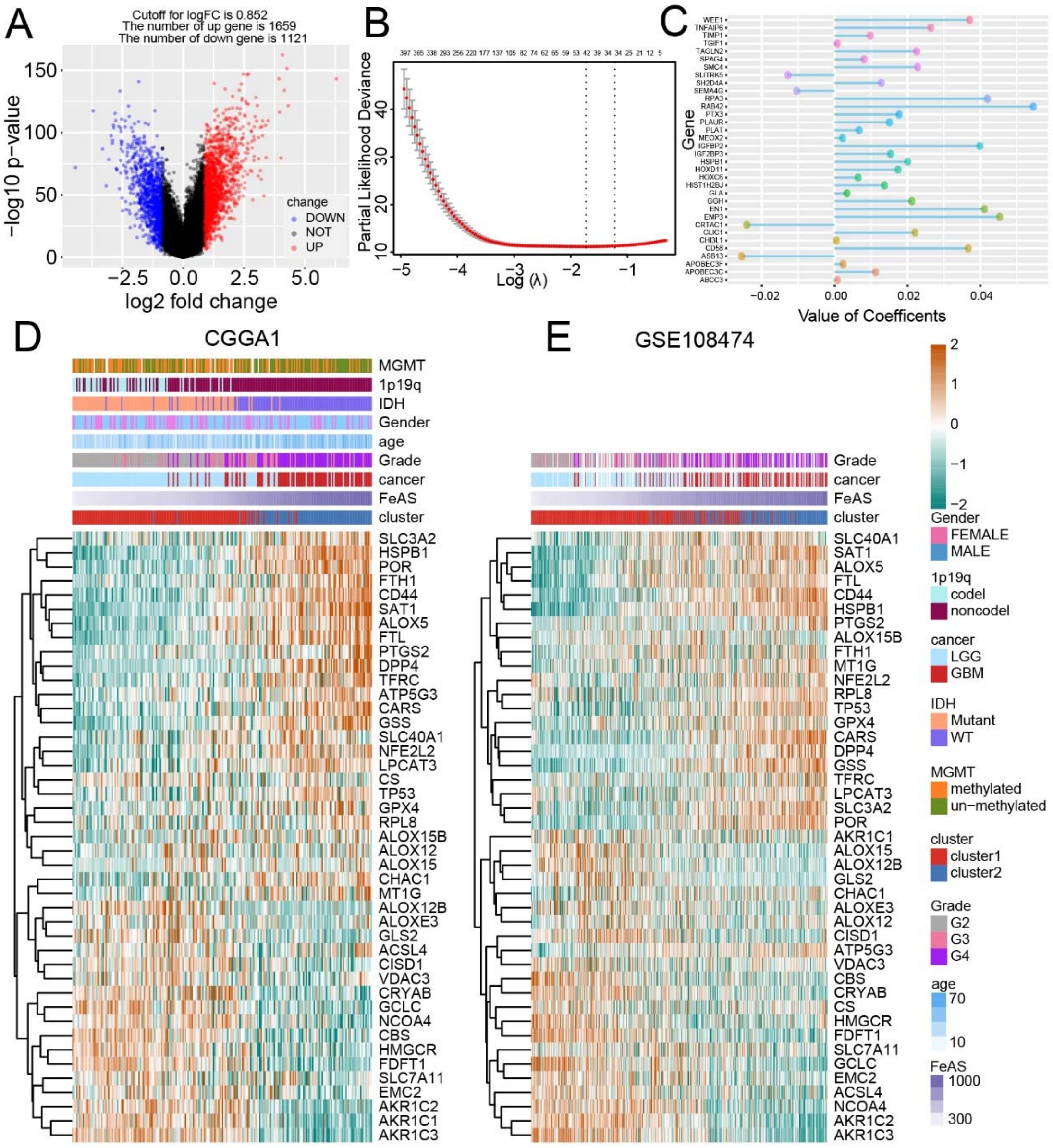

Supplementary Figure 3 | The construction of the FeAS model. (A) Differential expression genes between cluster1 and cluster2. (B) The elastic net analysis based on differential expression genes. (C) Results of the elastic net analysis. Ferroptosis related expression along with clinical features in the clustering model in the CGGA1 (D) and GSE108474 database (E).

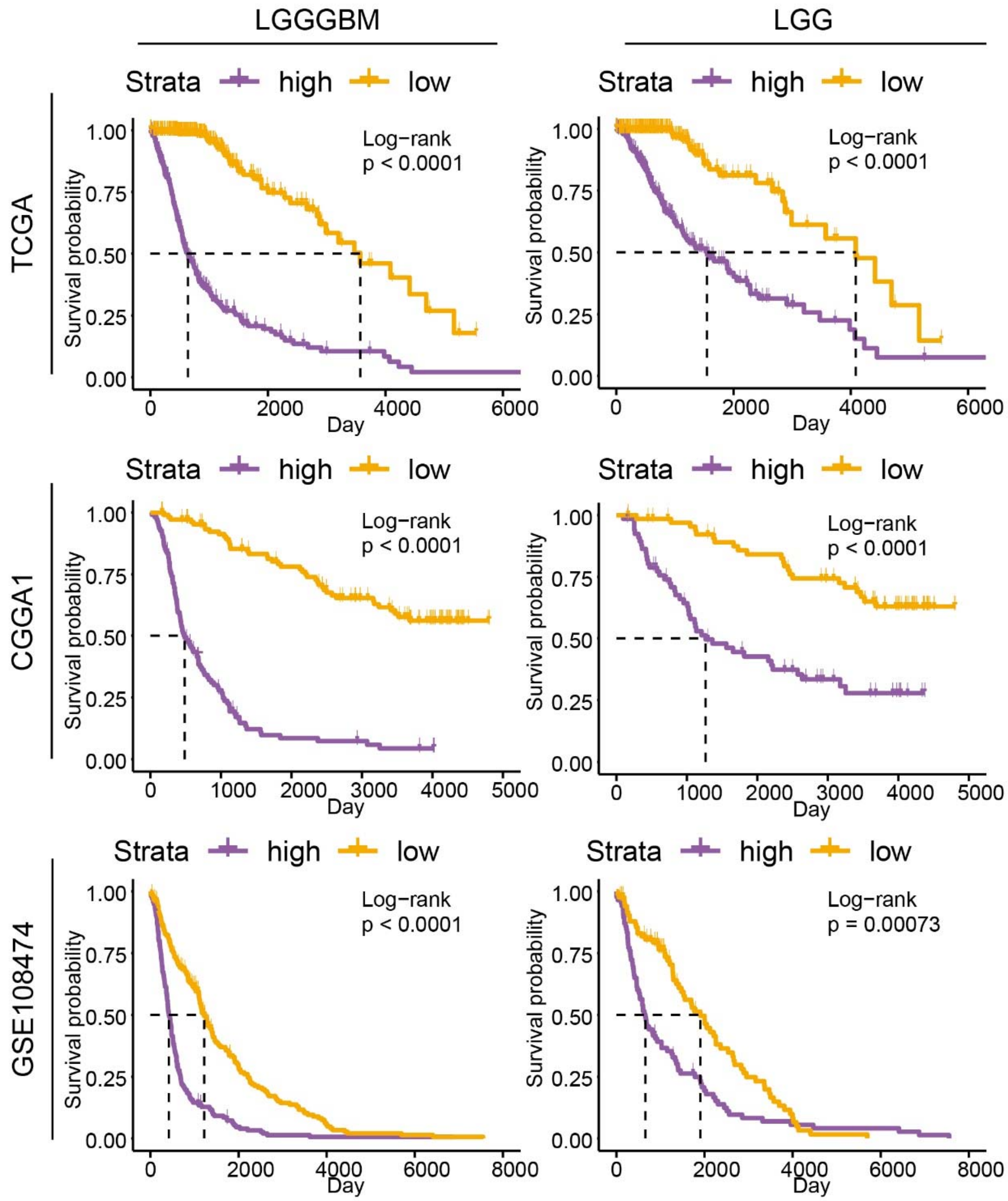

Supplementary Figure 4 | Overall survival analysis based on the FeAS model. High FeAS samples show worse survival outcome than low FeAS samples in the LGGGBM cohort (TCGA: P value < 0.0001; CGAA1: P value < 0.0001; GSE108474: P value < 0.0001). High FeAS samples show worse survival outcome than low FeAS samples in the LGG cohort (TCGA: P value < 0.0001; CGAA1: P value < 0.0001; GSE108474: P value = 0.00073).

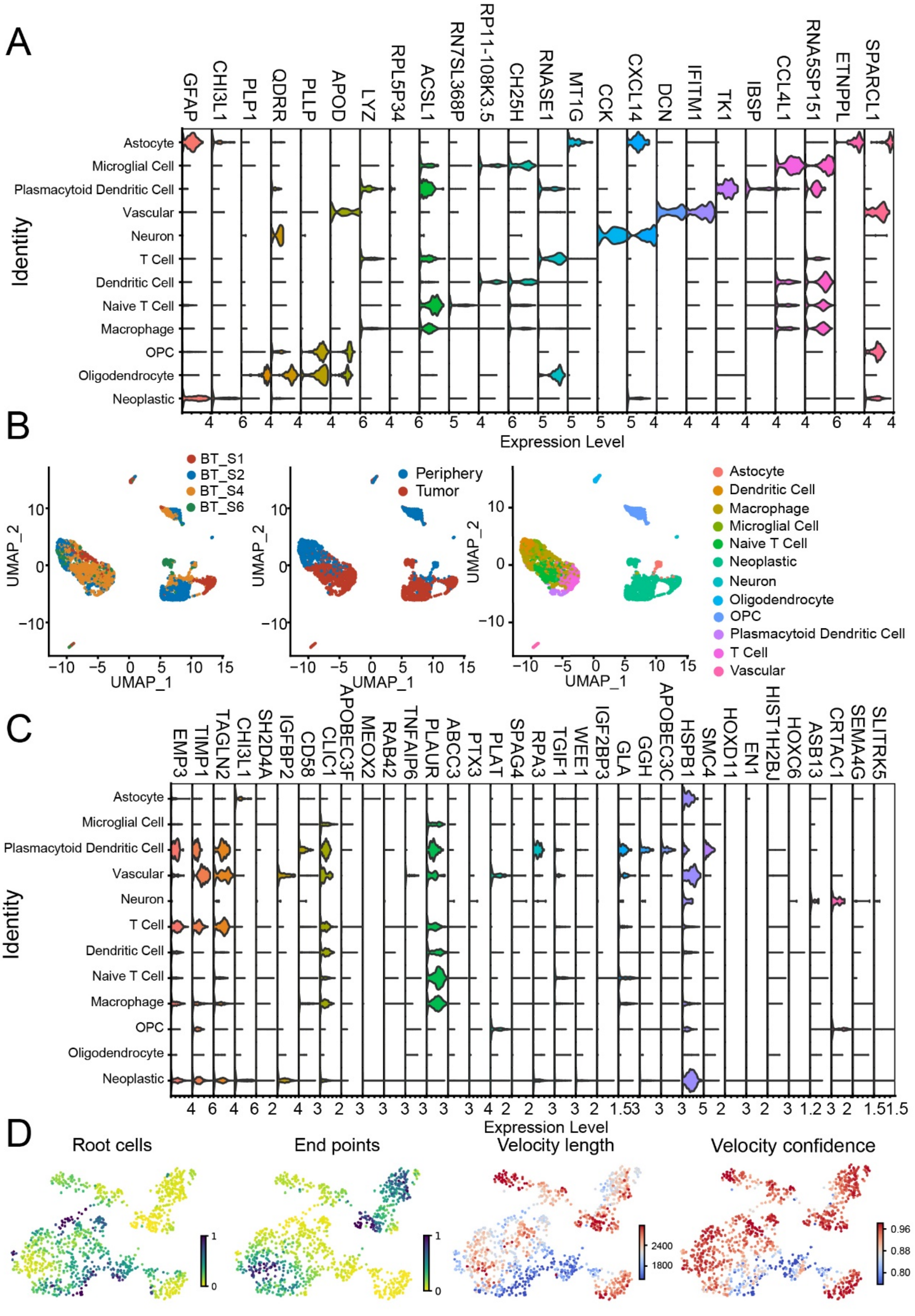

Supplementary Figure 5 | Details about the single cell RNA-seq analysis. (A) The distribution of the marker of each cell subtype. (B) The cell component of GSE84465. (C) Expression profile of the result of the elastic net analysis. (D) Details about the RNA velocity.

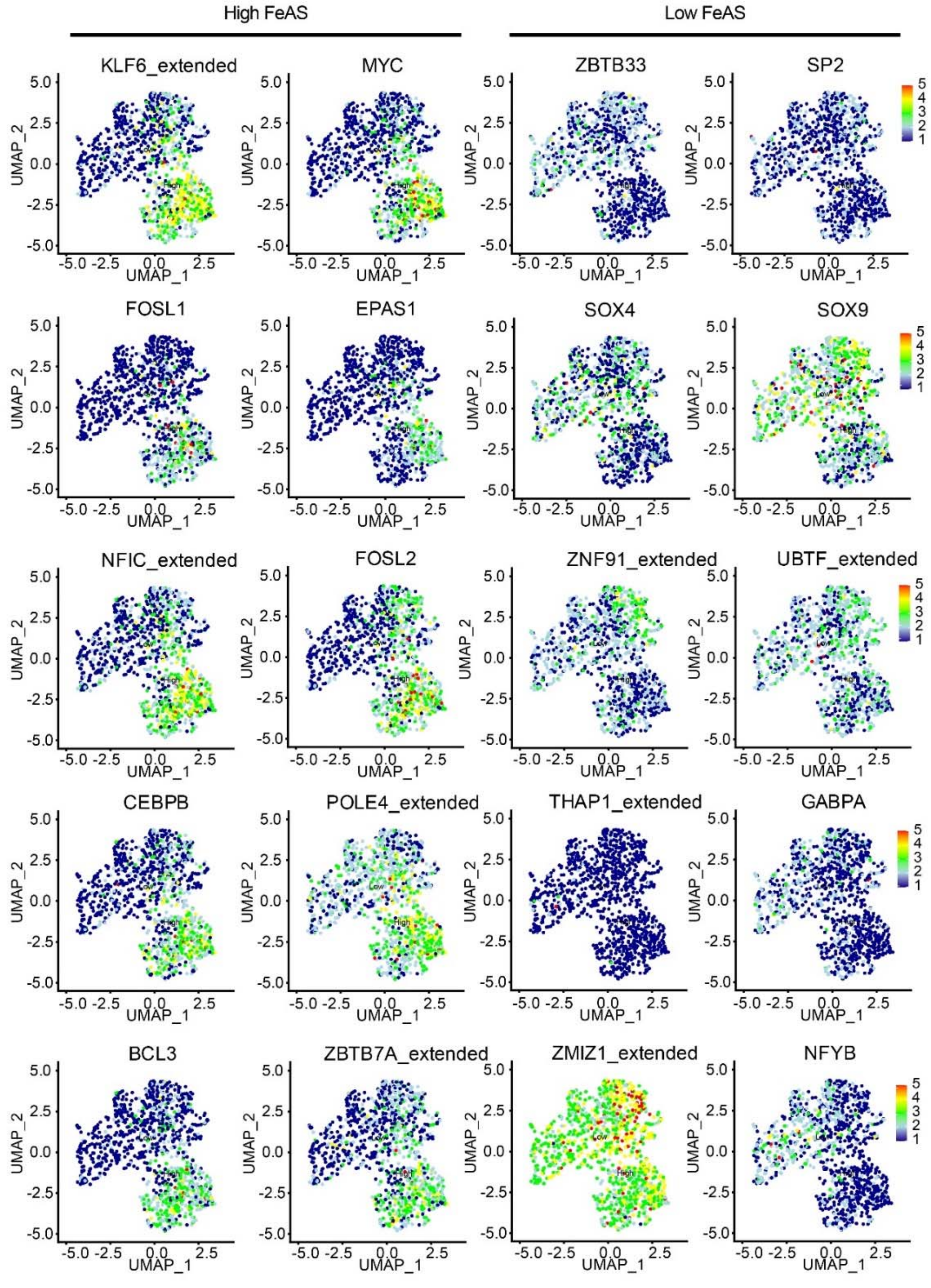

Supplementary Figure 6 | Top 10 transcription factor which enriched in high and low FeAS samples respectively. KLF6_extended, MYC, FOSL1, EPAS1, NFIC_extended, FOSL2, CEBPB, POLE4_extended, BCL3, ZBTB7A_extended are differential activated in high FeAS samples; while ZBTB33, SP2, SOX4, SOX9, ZNF91_extended, UBTF_extended, THAP1_extended, GABPA, ZMIZ1_extended, NGYB are active in low FeAS samples.

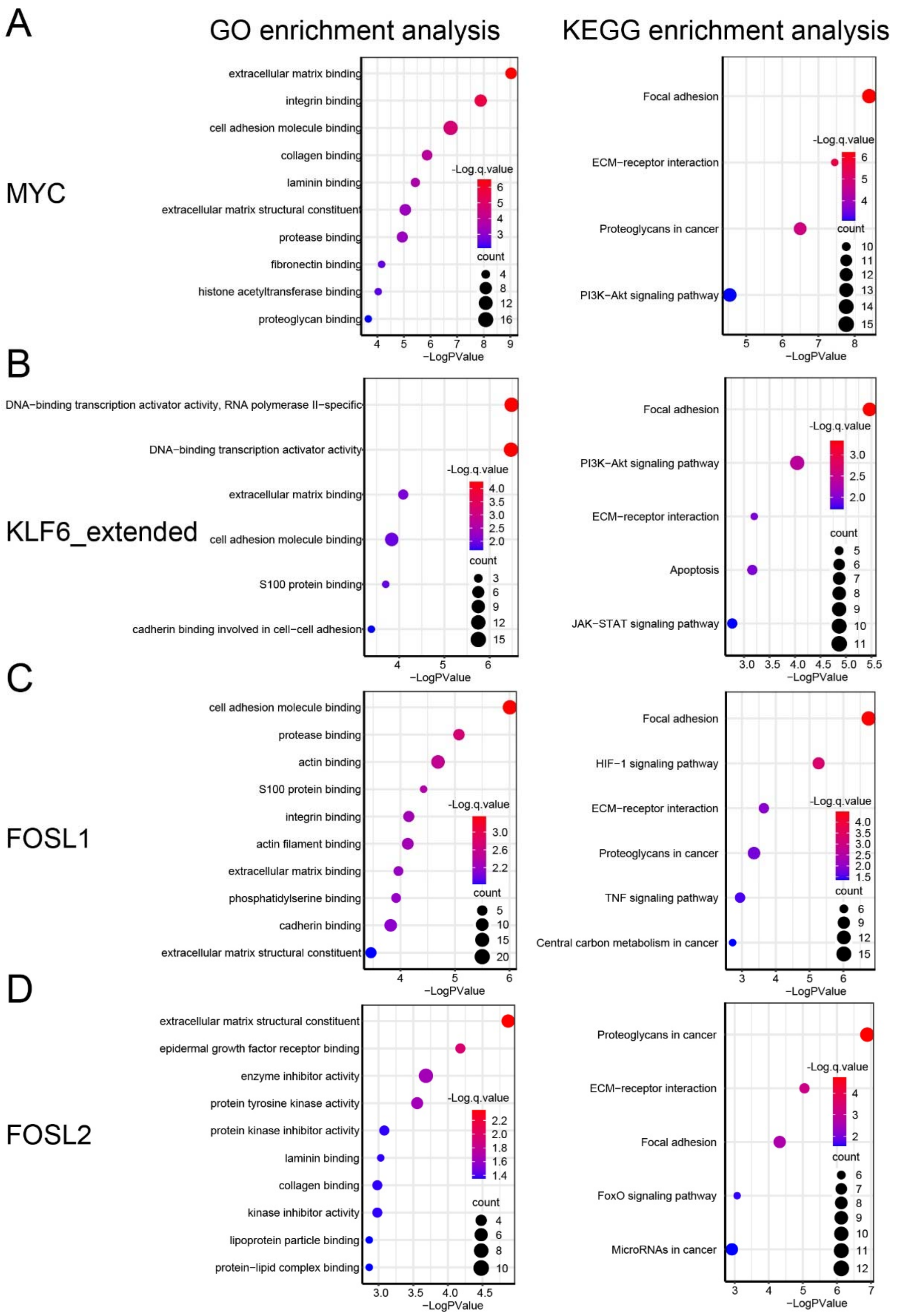

Supplementary Figure 7 | GO and KEGG enrichment analysis based on transcription factor from high FeAS GBM cells in single cell RNA-seq analysis. GO and KEGG enrichment analysis based on MYC (A), KLF6_extended (B), FOSL1 (C), FOSL2 (D).

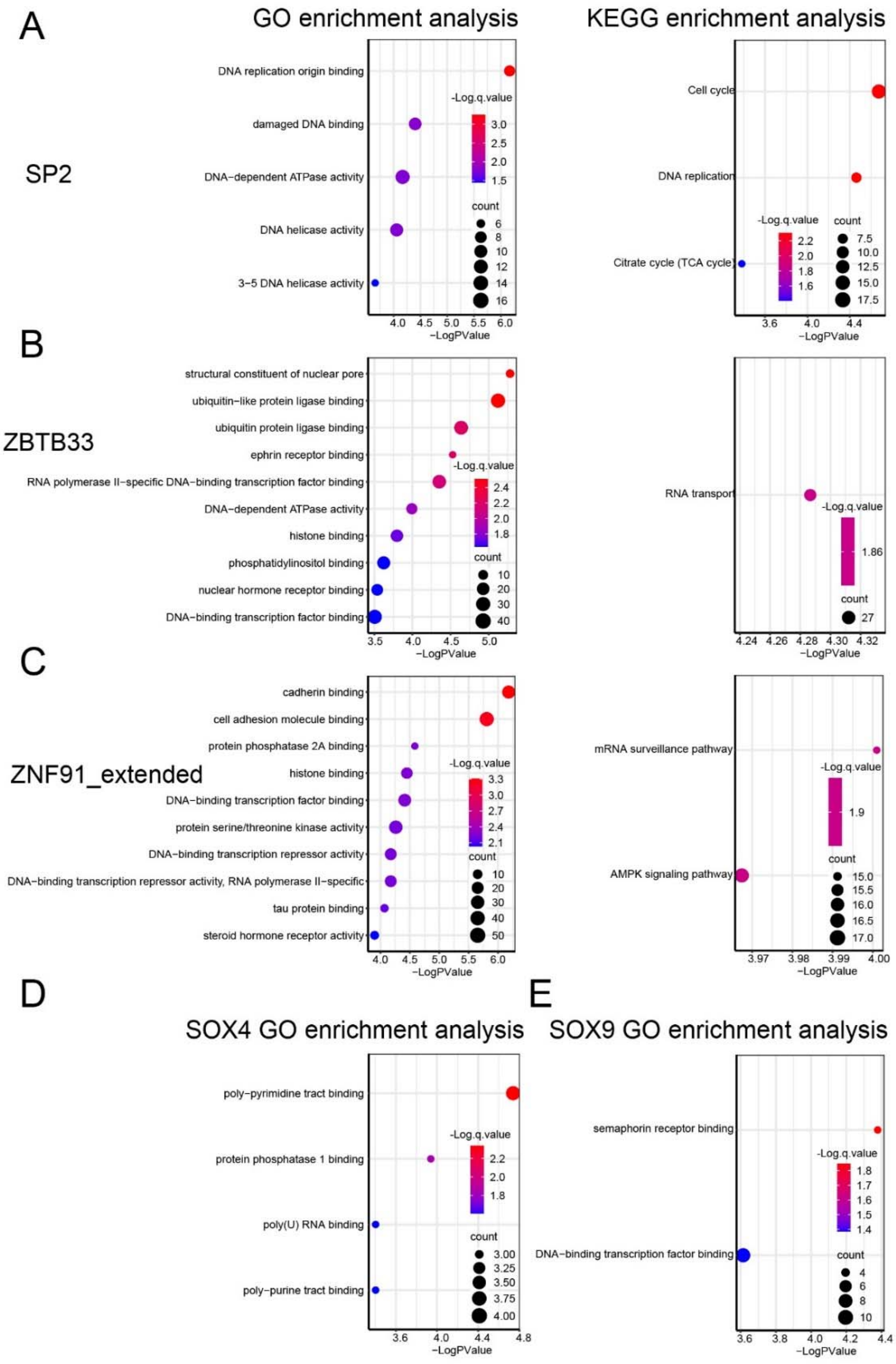

Supplementary Figure 8 | GO and KEGG enrichment analysis based on transcription factor from low FeAS GBM cells in single cell RNA-seq analysis. GO and KEGG enrichment analysis based on SP2 (A), ZBTB33 (B), ZNF91_extended (C). GO enrichment analysis based on SOX4 (D) and SOX9 (E).

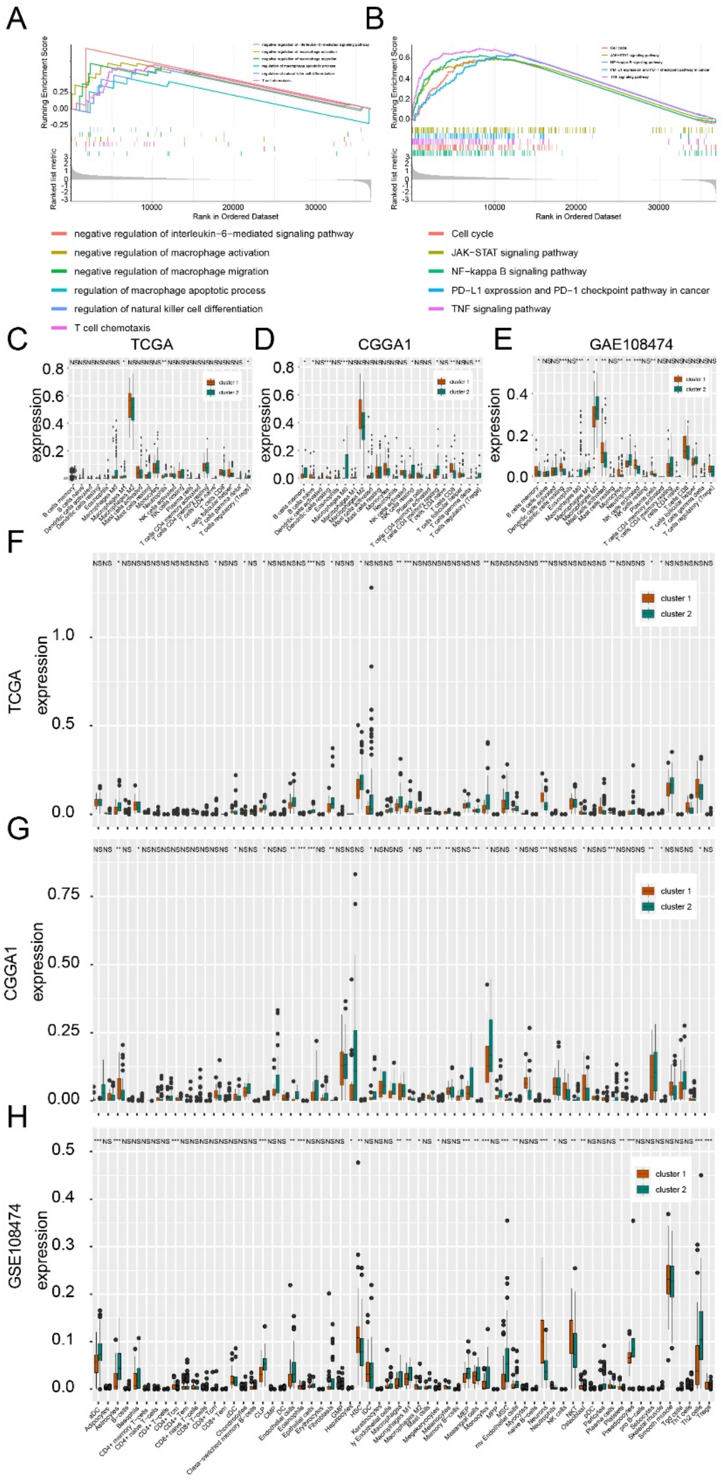

Supplementary Figure 9 | Biofunction prediction based on the cluster model. GO (A) and KEGG (B) enrichment analysis based on GSEA analysis. CIBERSORT and xCell algorithm are performed in TCGA (C, F), CGGA1 (D, G) and GSE108474 (E, H) databases respectively.

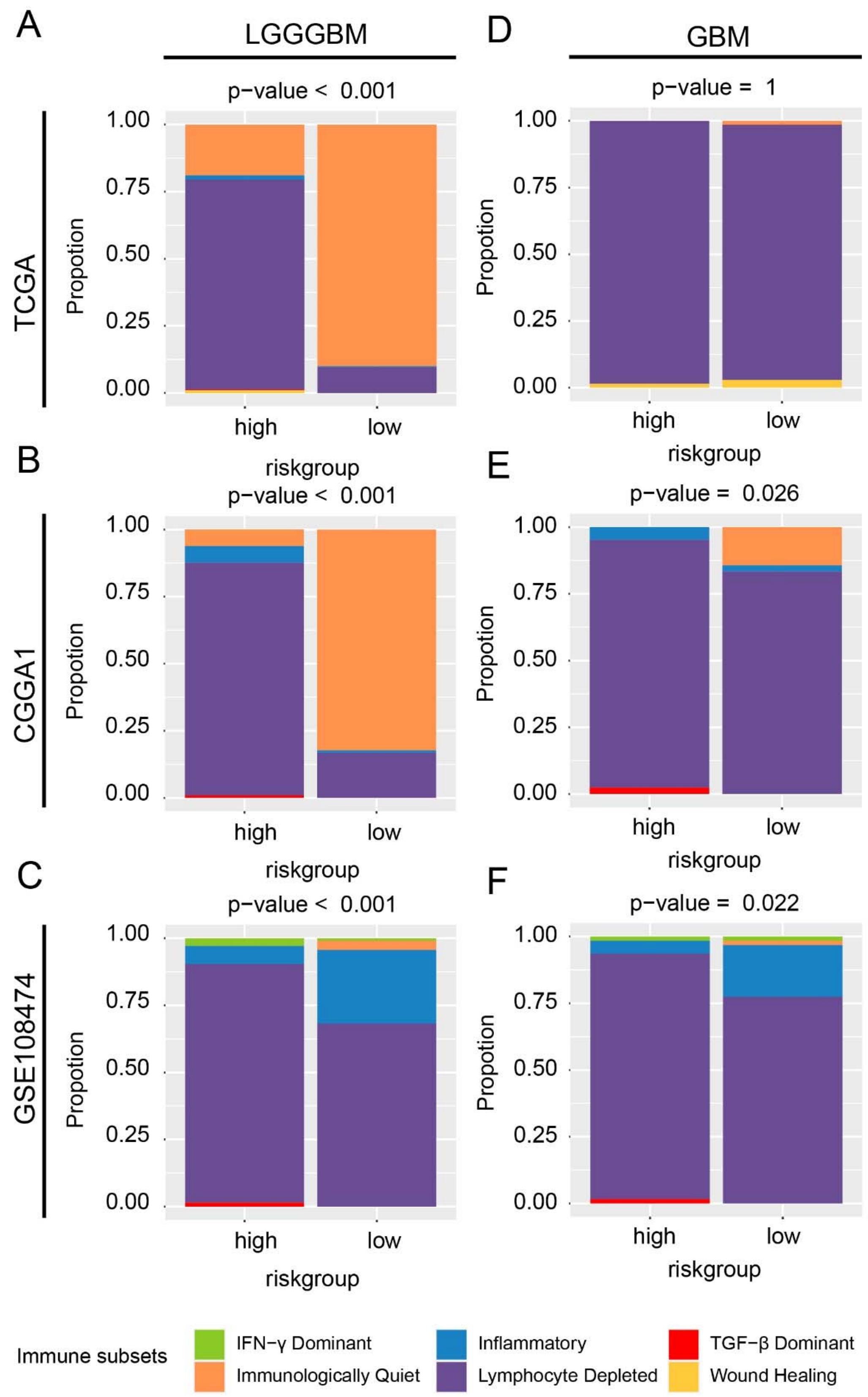

Supplementary Figure 10 | Tumor immune subset based on bulk RNA-seq analysis. Immune subset of gliomas from TCGA (A), CGGA1 (B), GSE108474 (). Immune subset of gliomas from TCGA (D), CGGA1 (E), GSE108474 (F).

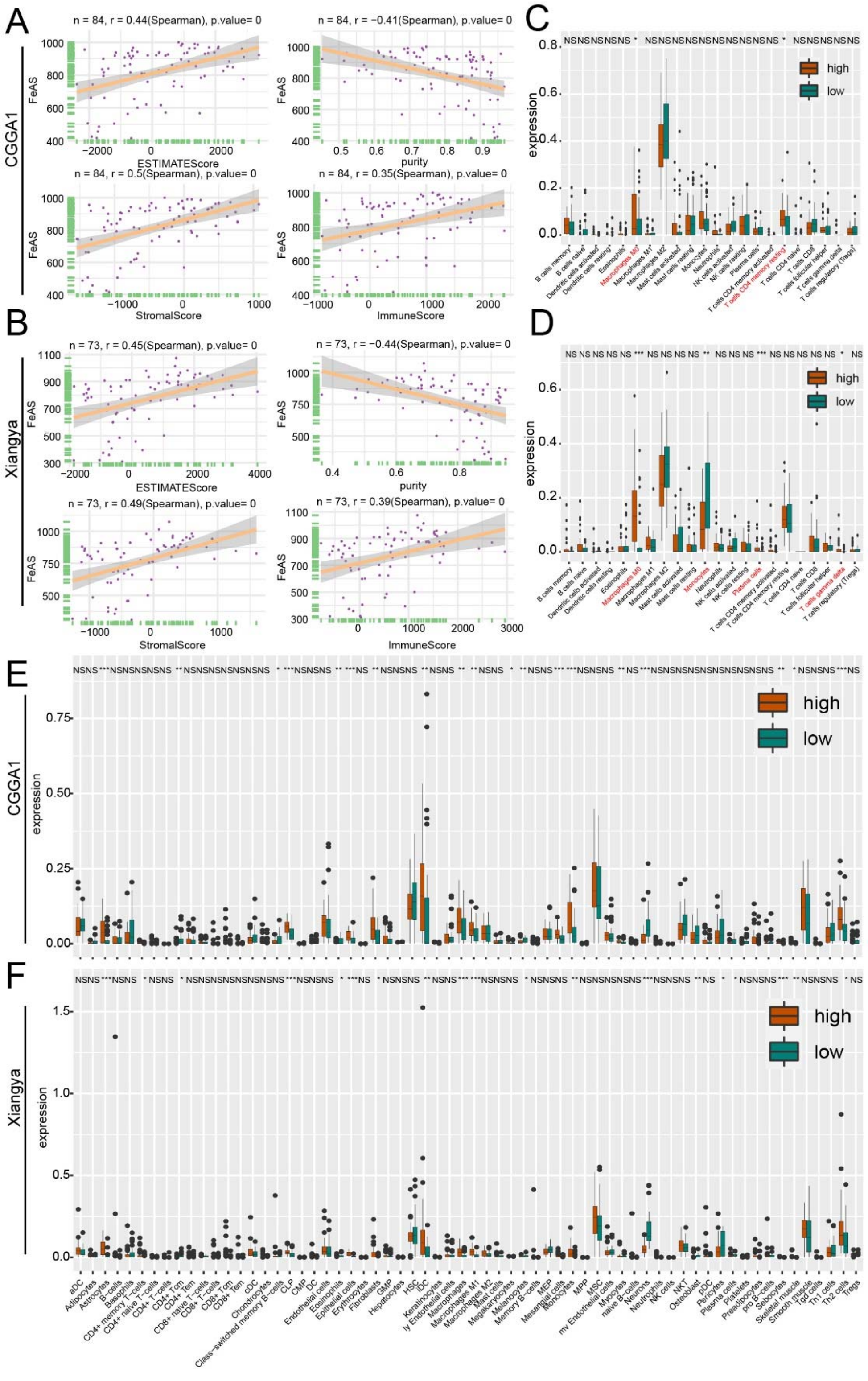

Supplementary Figure 11 | Tumor immune landscape based on bulk RNA-seq analysis in CGGA1 and Xiangya database. The ESTIMATE algorithm based on CGGA1 (A) and Xiangya (B) database. The CIBERSORT algorithm based on CGGA1 (C) and Xiangya (D) database. The xCell algorithm based on CGGA1 (E) and Xiangya (F) database.

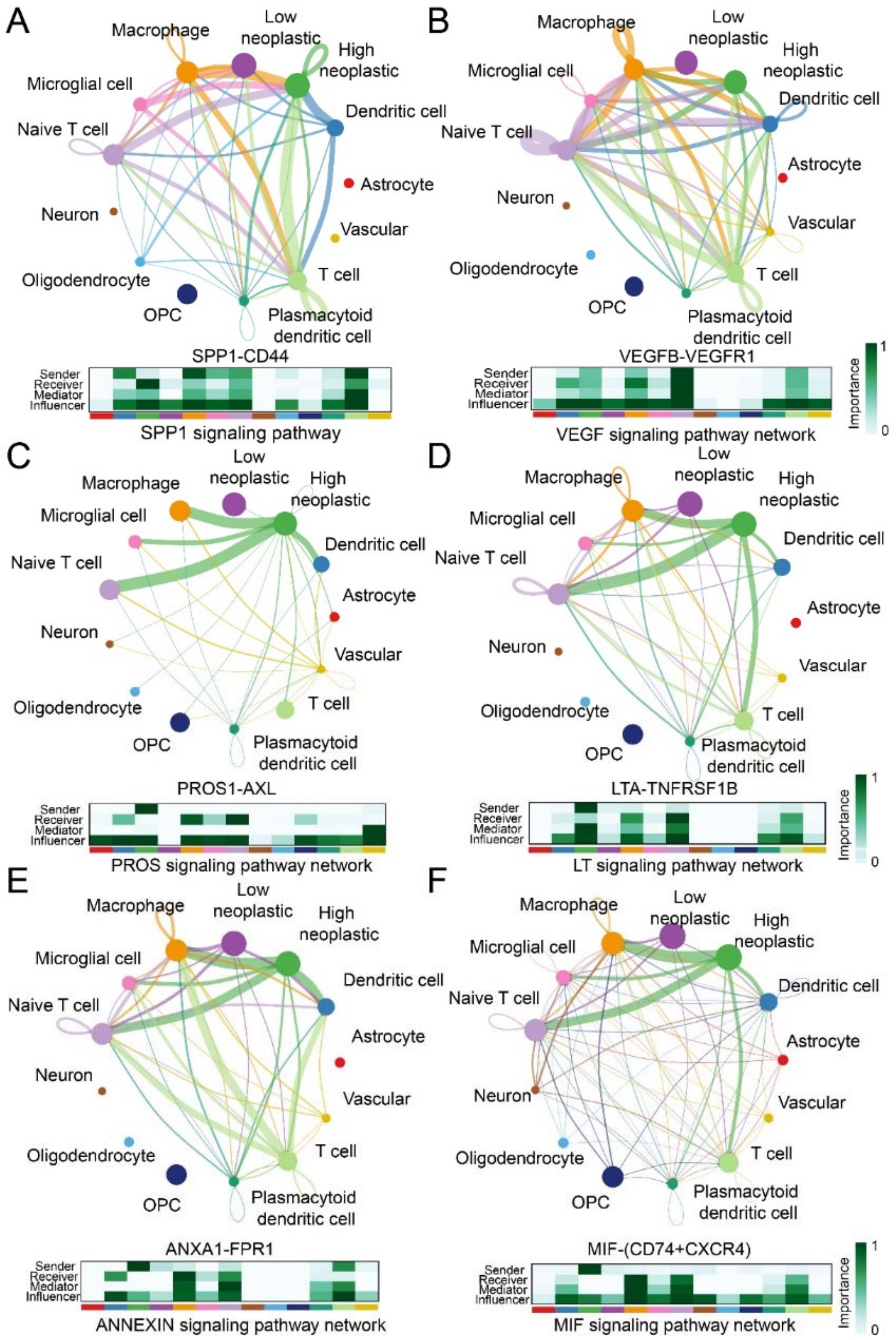

Supplementary Figure 12 | Ligand-receptor pairs difference between high and low FeAS samples. (A) High FeAS cells communicates with macrophage, microglial, naïve T cell and dendritic cells through SPP1-CD44. (B) High FeAS cells communicates with macrophage and T cell through VEGFB-VEGFR1. (C) High FeAS cells communicates with macrophage, microglial and naïve T cell through PROS1-AXL. (D) High FeAS cells communicates with macrophage, naïve T cell and T cell through LTA-TNFRSF1B. (E) High FeAS cells communicates with macrophage, naïve T cell and dendritic cells through ANXA1-FPR1. (F) High FeAS cells communicates with macrophage, microglial cell and naïve T cell through MIF-(CD74+CXCR4).
